# A universal co-solvent dilution strategy enables facile and cost-effective fabrication of perovskite photovoltaics

**DOI:** 10.1038/s41467-021-27740-4

**Published:** 2022-01-10

**Authors:** Hong Zhang, Kasra Darabi, Narges Yaghoobi Nia, Anurag Krishna, Paramvir Ahlawat, Boyu Guo, Masaud Hassan S. Almalki, Tzu-Sen Su, Dan Ren, Viacheslav Bolnykh, Luigi Angelo Castriotta, Mahmoud Zendehdel, Linfeng Pan, Sandy Sanchez Alonso, Ruipeng Li, Shaik M. Zakeeruddin, Anders Hagfeldt, Ursula Rothlisberger, Aldo Di Carlo, Aram Amassian, Michael Grätzel

**Affiliations:** 1 Laboratory of Photonics and Interfaces, École Polytechnique Fédérale de Lausanne, Lausanne, 1015 Switzerland; 2 Department of Materials Science and Engineering, and Organic and Carbon Electronics Laboratories (ORaCEL), North Carolina State University, Raleigh, NC 27695 USA; 3 Centre for Hybrid and Organic Solar Energy (CHOSE), University of Rome Tor Vergata, Rome, 00133 Italy; 4 Laboratory of Photomolecular Science, Institute of Chemical Sciences and Engineering, École Polytechnique Fédérale de Lausanne, Lausanne, 1015 Switzerland; 5 Laboratory of Computational Chemistry and Biochemistry, École Polytechnique Fédérale de Lausanne, Lausanne, 1015 Switzerland; 6 Kimia Solar Research Institute, Kimia Solar Company, Kashan, 87137-45868 Iran; 7 National Synchrotron Light Source II, Brookhaven National Laboratory, Upton, NY 11973 USA; 8 Department of Chemistry – Ångström Laboratory, Uppsala University, 751 20 Uppsala, Sweden; 9 ISM-CNR, Institute of Structure of Matter, National Research Council, via del Fosso del Cavaliere 100, 00133 Rome, Italy

**Keywords:** Materials chemistry, Materials for devices, Solar cells

## Abstract

Cost management and toxic waste generation are two key issues that must be addressed before the commercialization of perovskite optoelectronic devices. We report a groundbreaking strategy for eco-friendly and cost-effective fabrication of highly efficient perovskite solar cells. This strategy involves the usage of a high volatility co-solvent, which dilutes perovskite precursors to a lower concentration (<0.5 M) while retaining similar film quality and device performance as a high concentration (>1.4 M) solution. More than 70% of toxic waste and material cost can be reduced. Mechanistic insights reveal ultra-rapid evaporation of the co-solvent together with beneficial alteration of the precursor colloidal chemistry upon dilution with co-solvent, which in-situ studies and theoretical simulations confirm. The co-solvent tuned precursor colloidal properties also contribute to the enhancement of the stability of precursor solution, which extends its processing window thus minimizing the waste. This strategy is universally successful across different perovskite compositions, and scales from small devices to large-scale modules using industrial spin-coating, potentially easing the lab-to-fab translation of perovskite technologies.

## Abstract (web summary)

A universal strategy is reported for formation of very low concentration of perovskite precursor inks that yields high-efficiency photovoltaics while substantially reducing toxic waste generation, fabrication cost and process complexity, thus taking a step closer towards commercialization.

## Introduction

Solution-processed perovskite solar cells (PSCs) have attracted enormous attention due to the prospect of low-cost, easy fabrication of high-performance photovoltaic thin-films^1–6^. Despite the rapid increase of PSC efficiency from 3.8% in 2009 to 25.5% in 2020^7^, the prospect of commercialization remains bleak because of the presence of toxic lead components and the generation of toxic waste from the precursor solution during manufacturing^8^. Several research groups have tried to replace or reduce the lead usage with other less toxic metal elements (e.g., tin^9^, germanium^10^, and bismuth^11^), however, the stability and efficiency of lead-free or reduced content of lead PSCs are still far behind the state-of-the-art lead-based PSCs and the solvents used for processing are still toxic in nature. Other strategies have recently focused on preventing the leaching of toxic lead from solar cells, but these do not address the toxic waste generation^12^. Therefore, so far, the problem of toxic waste generation has not been addressed scientifically despite much of it residing in the formulation, storage, and processing steps. A conventional perovskite precursor solution is made of high purity lead halide salts, organic or inorganic ammonium salts, dissolved in toxic polar aprotic solvents (e.g., dimethylformamide (DMF), dimethylacetamide (DMAc), *N*-methyl-2-pyrrolidone (NMP), etc.)^8^ and other high-boiling point solvents such as DMSO in high concentration^5,13^. Although few green solvents have been developed to replace these toxic solvents, the resulting device performance is still not comparable with that of the DMF-based precursor^13^. In addition, these high purity perovskite raw materials (PbI_2_, formamidinium iodide (FAI), methylammonium iodide (MAI), etc.) are still very expensive (Supplementary Table 1), which increases the fabrication cost, and have a short shelf life due to aggregation.

Typically, a direct correlation between the thickness of the perovskite film and the precursor solution concentration has meant that fabrication of state-of-the-art PSCs requires a relatively high concentration (>1.4 M) of lead components^14,15^ to obtain prototypical thin films (i.e., thick, compact, highly crystalline, and pinhole-free) for photovoltaic devices^16^. Record efficiencies are currently achieved through a combination of spin-coating highly concentrated precursor solutions with an anti-solvent drip approach^13,17^. But one downside of most laboratory-based spin-coating of high concentration solutions is precursor waste generated during processing, which cannot be addressed by lowering the concentration of traditional precursors without also lowering film thickness and power conversion efficiency (PCE). Such high concentration perovskite solution with high viscosity and low evaporation rate create obstacles to typical scalable perovskite deposition methods in industrial applications, e.g., slot-die coating, spray coating, blade coating, ink-jet printing, roll-to-roll printing as well as industrial-based spin coating, by slowing down the continuous production rate and reducing opportunities for collection and reuse of excess solution. Another downside of concentrated solutions is their short shelf-life due to their propensity to aggregate and agglomerate, which increases toxic waste. Therefore, there is an urgent requirement to develop an eco-friendly strategy that can minimize the consumption of toxic materials by simultaneously reducing toxic waste during the processing step and by extending the shelf life of perovskite inks.

Here, we present a co-solvent dilution strategy that maintains high-quality perovskite films with very low concentration precursor solutions. This strategy substantially reduces the quantity of expensive raw materials in the perovskite precursor ink and reduces the toxic waste production by spin coating through two key routes: minimizing precursor loss during the processing of perovskite films and enhancing the lifetime and shelf-life of the inks by suppressing aggregation of precursor colloids. A PCE of over 24% for laboratory PSCs could be achieved with a co-solvent dilution to a level as low as 0.5 M. In addition, scalability of the co-solvent dilution strategy is tested via fabrication of perovskite solar modules (PSMs) with different sizes using industrial spin coating. The modules fabricated by co-solvent dilution strategy show higher PCEs and far better uniformity and reproducibility than modules prepared with conventional perovskite inks, whilst using a fraction of the precursor. Importantly, more than 70% toxic waste/solvent, perovskite raw material, and fabrication cost are projected to be reduced for module fabrication compared to the same modules made using conventional inks by industrial spin coating, and in doing so make spin coating a sustainable technique for medium scale manufacturing, for instance, for standalone modules or Si wafer-scale integration^18–21^. This work shows that through judicious selection of a greener co-solvent, we can significantly reduce the usage and waste of toxic solvents and perovskite raw materials, while also simplifying fabrication and cutting costs of PSCs.

## Results and discussion

### Proof of concept

The perovskite precursor solution is prepared with a conventional method^16^ (denoted as control; C). Then the judiciously designed co-solvent THF is added to the as-prepared perovskite precursor solution with different dilution ratios (denoted as a target; T) as shown in Supplementary Table 2 and Supplementary Fig. 1. Considering the precursor solubility and film quality (Supplementary Fig. 2), we choose tetrahydrofuran (THF) as an example of co-solvent to demonstrate our concept in this work with the following rationale: (i) a greener solvent with very low disability-adjusted life year (DALYs) per kg of a substance emitted among typical solvents of PSCs’ fabrication (Human health characterization factors extracted from USEtox 2.11)^22^; (ii) low boiling point and a very high vapor pressure (less energy and time required for drying) (Supplementary Table 3); (iii) comparatively weak coordination with lead (with respect to DMSO or DMF, see density functional theory (DFT) calculations and molecular dynamics (MD) simulations below); (iv) miscible with the parent solvents (DMF and DMSO) with less environmental impact during air emission of the solvent removed from the thin film^22^. We note that there are a few studies using THF as a co-solvent together with methylamine to prepare well-dissolved MAPbI_3_ perovskite precursor^23,24^. However, methylamine has been widely demonstrated to induce the formation of a non-perovskite phase upon reacting with FA-based perovskites^25,26^, which limits the application of previous strategies towards the fabrication of high-performance FA-based PSCs.

**Fig. 1:**
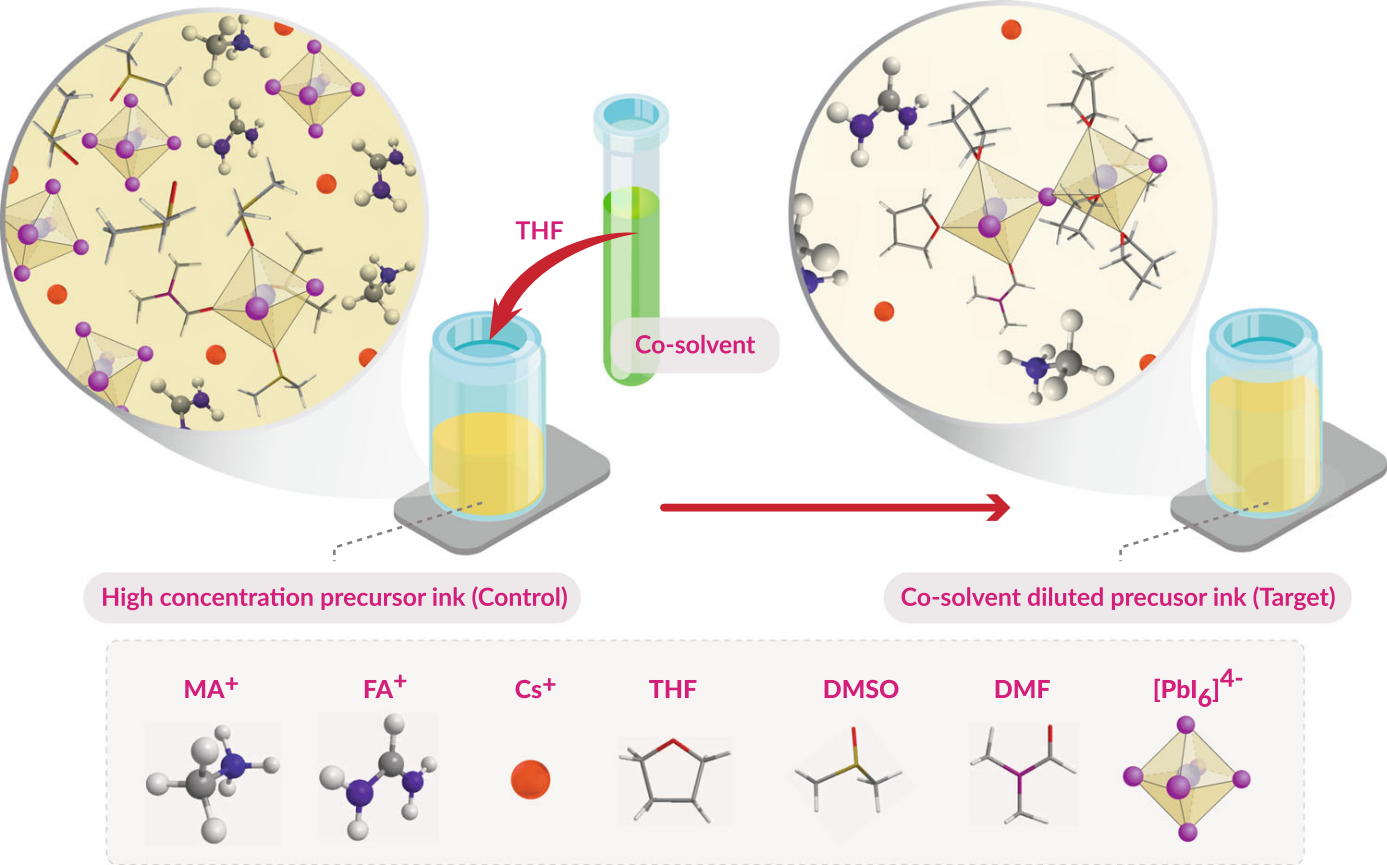
Schematic illustration of the co-solvent dilution strategy for perovskite deposition. Dilution of perovskite precursor inks by a cosolevnt, top part of the panel: schematic illustration of the dilution process using tetrahydrofuran as a cosolvent. Bottom part of the panel: chemical compounds and their structure employed in the precursor solution.

**Fig. 2:**
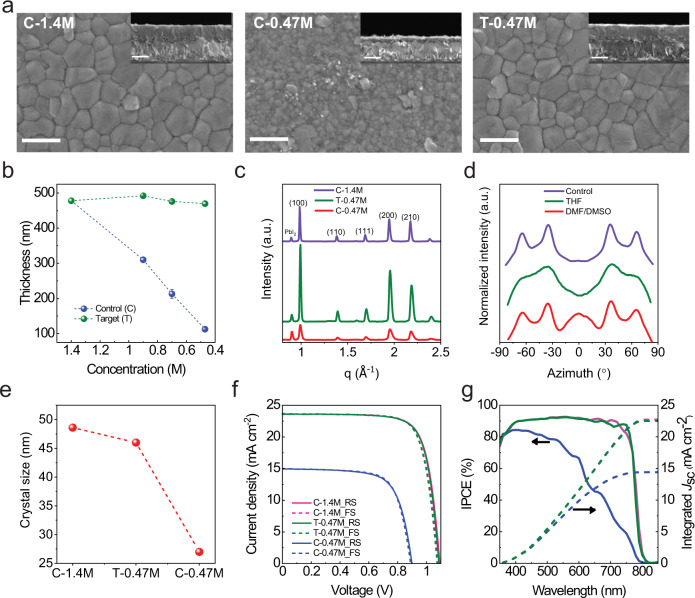
Effects of perovskite precursor concentration on the film properties. **a** SEM images of control and target triple-cation perovskite films with different precursor concentrations. The insets show cross-sectional SEM images. The scale bar is 500 nm. **b** The relationship between perovskite precursor concentration and film thickness. All films were prepared using identical coating conditions. The film thickness was measured by profilometry. **c** GIWAXS scattering intensity vs. *q*. **d** Azimuthally integrated intensity of the ring in *q* range of $1{\mathring{\rm A} }^{-1}$ assigned to (100) diffraction. **e** The change of crystal size of perovskite phase ($2{{{{{\rm{\theta }}}}}}=14^\circ$) vs. precursor composition. *J–V* curves (**f**) and IPCE spectra (**g**) of triple-cation PSCs are based on different precursor solutions.

As a proof of concept, we investigated our co-solvent dilution strategy with THF on the widely used triple-cation perovskites (Cs_0.05_(FA_0.9_MA_0.1_)_0.95_Pb(I_0.9_Br_0.1_)_3_) as illustrated in Fig. 1. The control precursor solutions were dissolved in DMF/DMSO (4/1, v/v) mixture with various concentrations viz.,1.40, 0.93, 0.70, and 0.47 M. For our target samples, the high concentration 1.40 M control precursor solution was diluted with different volumes of THF (i.e., dilution of 200 v% THF obtains a concentration of 0.47 M). To simplify the labeling, 1.4 M control solution, 200 v% DMF/DMSO, and 200 v% THF co-solvent diluted solution are hereafter denoted as C-1.40, C-0.47, and T-0.47 M, respectively. The perovskite films were fabricated with the above-mentioned precursor solutions using the widely adopted one-step spin-coating with the antisolvent drip method (see details in Methods). From scanning electron microscopy (SEM) top-view images in Fig. 2a, we observe that the morphology in C-1.40 M showed compact features of large grains. However, the C-0.47 M film morphology is poor containing small grains, poor surface coverage, and pinholes, whereas in the T-0.47 M sample the morphology is extremely compact with large and homogeneous grain size. The cross-section SEM images in the insertion of Fig. 2a reveal that C-1.4 M film has a thickness of ~480 nm which is typical for such concentration and upon dilution from C-0.93 to C-0.47 M film, the thickness is drastically reduced to ~310 and ~210 nm, respectively, which is detrimental for the light-harvesting capacity (Supplementary Fig. 3). In contrast, by diluting from T-0.93 to T-0.35 M with our co-solvent strategy, there is no meaningful reduction in thickness (Fig. 2b). Grazing-incidence wide-angle X-ray scattering (GIWAXS) and X-ray diffraction (XRD) measurements were performed to determine the crystal structure and orientation in these samples (Supplementary Fig 4a and Supplementary Fig. 5a). GIWAXS 2D scattering maps were converted into 1D profile graphs by taking the circular average which possesses the crystalline information and results are shown in Fig. 2c. For C-0.47 M film, the diffraction intensity decreases dramatically, consistent with excessive material loss (Supplementary Fig. 4b and Supplementary Tables 3 and 4). In contrast, the diffraction features of the T-0.47 M film show similar peak broadening compared to C-1.4 M in XRD and in GIWAXS. The (100) perovskite diffraction powder ring at $q=1{\mathring{\rm A} }^{-1}$ was integrated azimuthally to assess the texture of the (100) planes (Fig. 2d). The C-1.4 M sample shows four indistinct diffraction regions at ${\pm}\!35.9^{^\circ }$ and ${\pm}\!{65.8}^{^\circ }$ which is consistent with previous reports on this perovskite composition^27^. Dilution with THF resulted in a more uniform powder texture distribution which we attribute to rapid solvent evaporation and phase transformation dynamics. Figure 2e shows the crystal size comparison extracted from Scherrer analysis on the (100) perovskite diffraction from XRD and GIWAXS measurements (Supplementary Figs. 4b and 5b and Supplementary Tables 3 and 4). T-0.47 M films achieve a slightly smaller crystallite size than C-1.4 M, consistent with a larger nucleation rate, whereas C-0.47 M yields a significantly smaller crystal size.

To check the impact of co-solvent dilution on the opto-electronic properties of perovskite film, we performed time-resolved photoluminescence (TRPL) decay as shown in Supplementary Fig. 6. We performed our experiments at low fluences (<5 nJ/cm^2^) so that bimolecular and Auger recombination is negligible and the TRPL after *t* > 100 ns is dominated by a monoexponential decay. Considering bulk recombination, we derive for the T-0.47 M film a first-order rate constant for nonradiative carrier recombination of *k*_1_ = 0.97 × 10^6^ s^−1^ corresponding to a lifetime of *τ* = 513.8 ns, while the respective values for the C-1.40 M film are *k*_1_ = 1.08 × 10^6^ s^−1^ and *τ* = 463.0 ns. In addition, PSCs with different control and target solutions were fabricated (as detailed in Methods) and compared to investigate the dilution effects on the photovoltaic performances and device stability. The PCE of the devices decreased from 19.55% to 9.43% due to the loss of absorption when the control precursor solution concentration was decreased from 1.40 to 0.47 M as shown in Fig. 2f, g. The device fabricated with T-0.47 M showed an impressive PCE of over 19% (Table 1 and Supplementary Fig. 7) with comparable operational stability as C-1.40 M based devices (Supplementary Fig. 8). This high PCE after co-solvent dilution with co-solvent, taken together with the SEM, TRPL, incident photon-to-current conversion efficiency (IPCE) data, and operational stability test, demonstrate that there is no reduction in the quality or thickness of perovskite films prepared by the co-solvent dilution strategy.

**Table 1:** Photovoltaic performance of PSCs based on different perovskite compositions with and without co-solvent dilution.

Perovskite composition	Precursor inks^a^	Sweep	*V*_oc_ (V)	*J*_sc_ (mA/cm^2^)	FF (%)	PCE (%)^b^	Avg. PCE (%)
Cs_0.05_(MA_0.1_FA_0.9_)_0.95_Pb(I_0.9_Br_0.1_)_3_	C-1.4 M	RS	1.09	23.65	75.70	19.55	19.38 ± 0.18
		FS	1.08	23.68	75.30	19.27	19.09 ± 0.19
	T-0.47 M	RS	1.08	23.58	76.20	19.47	19.19 ± 0.16
		FS	1.07	23.66	76.20	19.32	18.92 ± 0.21
MAPbI_3_	C-1.2 M	RS	1.12	22.57	78.20	19.75	18.98 ± 0.40
		FS	1.11	22.85	67.70	17.12	16.00 ± 0.97
	T-0.4 M	RS	1.13	22.55	76.70	19.49	19.06 ± 0.37
		FS	1.11	22.63	71.70	18.06	17.05 ± 0.41
FA_0.97_MA_0.03_PbI_2.91_Br_0.03_	C-1.5 M	RS	1.17	25.52	80.60	24.13	23.84 ± 0.17
		FS	1.15	25.45	77.90	22.88	22.64 ± 0.52
	T-0.5 M	RS	1.17	25.10	81.60	24.02	23.80 ± 0.17
		FS	1.16	25.16	78.12	22.80	22.64 ± 0.24

^a^C refers to control; T refers to target with 200 v% co-solvent dilutions.

^b^The champion device.

### Co-solvent dilution mechanism

**Fig. 3:**
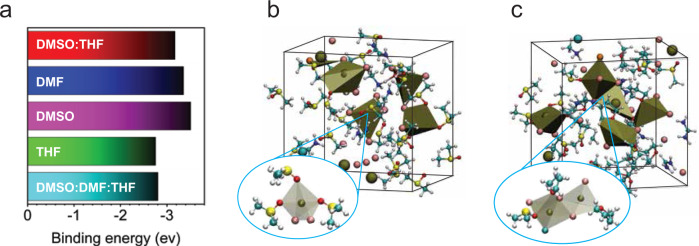
Solvent-solute interactions. **a** Average binding energies of Pb^2+^ ions with solvents and their mixtures (evaluated on the tetracoordinated complexes shown in Supplementary Fig. 9). **b**, **c** Ab initio MD simulations of perovskites precursors in **b** DMSO and **c** THF/DMSO mixture after ~15 ps. All species are shown with balls and sticks representations. Pb ions are shown with golden color, I with pink color, Cs with greenish-blue, Br with orange, C with light blue, N with dark blue, O with red, S with yellow and H atoms with white color. In the **b** and **c** panels, lead coordination polyhedra are shown within a 3.6 Å cutoff.

To validate the co-solvent dilution mechanism, we employ a combination of experimental and computational methods, including DFT calculations, ab initio MD simulations as well as in-situ characterization of solution-processing. We first investigated the solvent-solute interaction by DFT calculations. We calculated the interaction energies of Pb^2+^ with pure DMSO, DMF, THF, and their mixtures for prototypical tetracoordinates complexes (Supplementary Fig. 9). We find that the interaction energy of Pb^2+^ with THF is considerably lower than with DMSO and DMF, as illustrated in Fig. 3a. We also find that an intimate mixture with THF can significantly decrease the average interaction energy of Pb^2+^ with, solvent molecules which we anticipate may have implications on the conversion chemistry, that is, if some THF remains coordinated, the conversion to the perovskite phase might be ameliorated, as will be demonstrated later.

To further understand the finite temperature solvation effects at the pre-nucleation stage, we performed ~15 ps ab initio MD simulations of solute-solvent mixtures without and with THF. Simulation details are presented in the section of Methods (Computational methods). To evaluate the effect of co-solvent dilution on the solvent-solute interaction, we use DMSO as a model system in the following discussion. As shown in Fig. 3b, we see that both Pb^2+^ and monovalent cations (FA^+^ and MA^+^) are highly coordinated with oxygen atoms of DMSO molecules, forming individual small clusters in the case of perovskite precursor in pure DMSO. This can be expected due to the high interaction energies with DMSO (see Fig. 3a). However, in pure THF, we find that the Pb-I edge-sharing clusters start to form during the MD simulations (Supplementary Fig. 10). This can also be expected due to the weaker bonding between THF and Pb ions because of the low donor number (D_N_) of THF (low-D_N_ solvents coordinate less strongly with Pb^2+^)^28^ and can explain that precursor complexes are not able to dissolve in pure THF (see Supplementary Fig. 11). Interestingly, we find a mixed behavior in the DMSO/THF mixed system with the formation of small mixed Pb-I-solvent clusters and large Pb-I edge-sharing clusters (Fig. 3c). These smaller solute-solvent clusters can also act as nucleation centers^29^. Similar behavior was also observed in the mixtures of DMSO/DMF/THF (Supplementary Fig. 12). One of the main insights from these simulations is that the addition of a co-solvent, like THF, can be used to tune the solvent-solute interaction strength with a direct impact on the subsequent conversion and crystallization process of the perovskite phase, as will be discussed below.

**Fig. 4:**
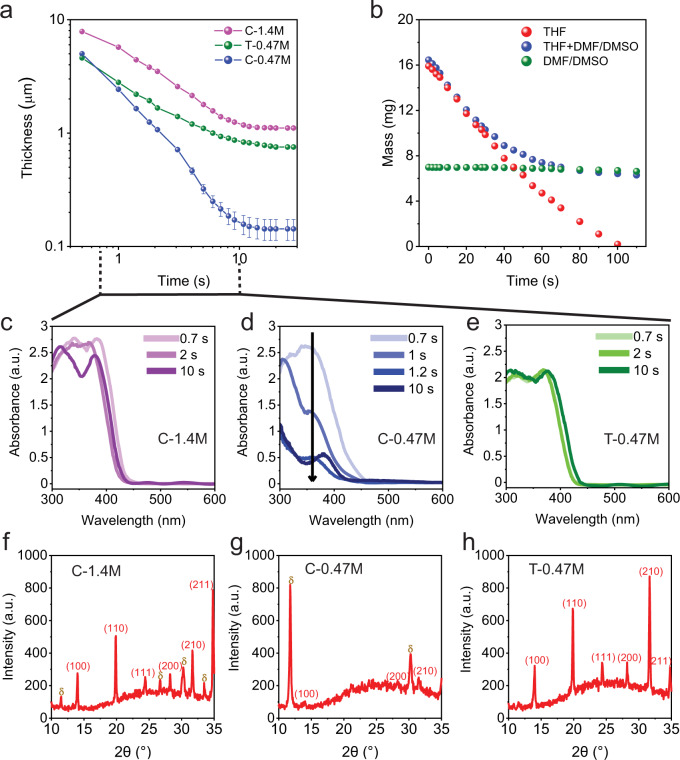
In situ UV–vis investigations and solvent evaporation rate measurement. **a** Thinning behavior of the solutions. **b** Solvent evaporation rate of THF, DMF/DMSO, and the mixture of these two. **c**–**e** Time-evolution of the solution absorbance in UV region during spin. **f**, **g** X-ray diffraction on the as-cast films.

We performed in situ UV–vis transmittance measurements during spin coating to investigate the evolution of solution thickness, as well as monitor the metallate precursor (UV absorbance) and perovskite phase formation (visible spectrum absorbance)^30,31^. Transmittance spectra were taken starting 0.5 s after spin coating and show interference oscillations (Supplementary Fig. 13) indicating a liquid precursor (sol) film whose thickness is rapidly decreasing, as shown in Fig. 4a. The wet sol films thin from ~5 to 8 μm to a steady-state thickness of ~1.1 μm for C-1.4 M, ~0.7 μm for T-0.47 M, and ~0.15 μm for C-0.47 M by the 20 s mark for anti-solvent drip. According to these measurements, the choice of co-solvent dilution strategy clearly dictates the steady-state thickness of the precursor film and strongly influences the solution thinning dynamics. Next, we seek to demonstrate the thinning behavior of T-0.47 M vs. C-0.47 M is due to THF’s rapid evaporation rate. To do so, we cast 20 µl of 200 v% THF-diluted DMF/DMSO mixture, a ~6.67 µl of DMF/DMSO mixture (volume of DMF/DMSO in 200 v% THF dilution) and 20 µl of pure THF onto a mass balance, and monitored the mass change vs. time as shown in Fig. 4b. The solvent mixture appears to follow Raoult’s law of mixtures whereby THF is removed from the mixture at the same rate as pure THF. The rapid THF evaporative loss increases the concentration and viscosity of the precursor solution of T-0.47 M compared to C-0.47 M, which disrupts its liquid-like characteristics, including ejection, outflow, and related waste during spin coating.

To further prove this point, we directly monitor the rate of absorbance decrease of precursor metallates in situ during solution processing. In Fig. 4c–e, we show the absorbance spectra for the three precursor solutions at multiple times ranging from 0.7 s (the earliest measurable transmittance spectrum) to 10 s during spin coating. According to these measurements, the absorbance signal drops sharply during the first 2 s of spin coating of C-0.47 M (Fig. 4d), indicative of continued precursor loss and waste, whereas the change is less pronounced for C-1.4 M (Fig. 4c) and completely absent for T-0.47 M (Fig. 4e). THF dilution is excellent at preserving the precursor absorbance levels, and spectral features, constant, proving that our co-solvent dilution strategy preserves the solution-cast metallate precursor on the substrate. Based on these observations, it is clear that the outflow step, responsible for precursor loss and toxic waste generation during spin-coating, can be significantly mitigated by our co-solvent dilution strategy.

We have investigated the phase transformation and crystallization of the sol film upon anti-solvent drip to assess the influence of co-solvent dilution strategy on the crystallization behavior. First, the investigation into the phase transformation of the sol film into perovskite upon antisolvent drip was performed by in situ UV–vis absorbance (Supplementary Fig. 14). The absorbance was normalized to the amount of precursor solute based on knowledge of the sol and final solid thickness after annealing and traced at a single wavelength. The absorbance vs. time data reveals C-1.4 and T-0.47 M to be quantitatively similar, indicating nominally similar phase transformations of the perovskite (Supplementary Fig. 14e). Nevertheless, the co-solvent appears to influence the precursor conversion chemistry when looking at the phase distribution just after the anti-solvent drip. Figure 4f–h shows the XRD measurements immediately after solution processing and anti-solvent drip in the N_2_ environment and prior to thermal annealing. The C-1.4 M sample exhibits both perovskite and non-perovskite hexagonal phases, while THF dilution promotes perovskite phase formation exclusively in as-cast films. In contrast, dilution with DMF/DMSO results in the non-perovskite phase becoming dominant, which because the excess of DMSO could accelerate the transformation of the black phase to non-perovskite phases^5^. This observation is supported by DFT and MD simulations, which predict a moderate weakening of metallate–DMSO interactions in the presence of THF. The “moderate” effect of the co-solvent is critical, in our view, as it would otherwise perturb delicate metallate-solvent interactions or upend the precursor chemistry which underpins the conversion and phase transformation of hybrid perovskites in the presence of DMSO/DMF solvents. In many ways, the failure of dilution in DMSO/DMF is a case in point as it seems to alter the precursor conversion chemistry and phase transformation behavior upon anti-solvent drip (Supplementary Fig. 14).

### Universality, scalability, and sustainability analysis

**Fig. 5:**
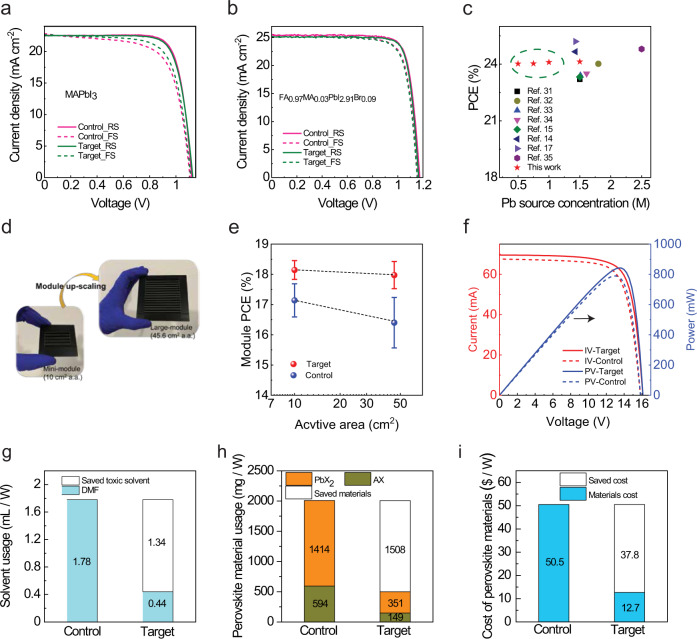
Scalability and sustainability. *J-V* curves of PSCs based **a** MAPbI_3_ and **b** FA_0.97_MA_0.03_PbI_2.91_Br_0.09_ perovskites. **c** Comparison of PCE in the literature and this work is based on different co-solvent dilution ratios. **d** Photograph images of the up-scaling program via fabrication of mini- and large perovskite modules by the co-solvent dilution strategy (Photo credit: Narges Yaghoobi Nia). **e** Statistic overview on the reproducibility of the co-solvent dilution versus control high concentrated modules influencing by the module active area. **f** *I–V* curves of champion large perovskite modules fabricated with high concentration control percussor and co-solvent diluted precursor. **g**–**i** Cost and waste generation analysis for module fabrication with and without co-solvent dilution. PbX_2_ = PbI_2_ + PbBr_2_; AX = FAI + MABr. The material cost includes the cost of all solvents and perovskite materials used.

We took the view that the co-solvent strategy’s moderate effects on lead-solvent interactions mean it should be universally applicable across other cation and halide compositions and other judiciously selected co-solvents, as well as scalable to large-area perovskite modules. Firstly, we employ our strategy for the widely investigated MAPbI_3_ perovskite as illustrated in Supplementary Fig. 15. The MAPbI_3_-based PSCs fabricated through the co-solvent dilution strategy of 200 v% THF dilution show a similar photovoltaic performance as that of higher concentration (1.2 M) control (Fig. 5a, Table 1, Supplementary Figs. 15h and 16). For the state-of-the-art PSCs based on double-cation and double-halide (I^−^, Br^−^) perovskite (FA_0.97_MA_0.03_PbI_2.91_Br_0.09_) in our group, the control device with 1.5 M concentration shows a high PCE of 23.84 ± 0.17%. By using our co-solvent dilution strategy, we achieve a similar quality of perovskite film as that of the conventional high concentration precursor as depicted in Supplementary Fig. 17. Importantly, the resulting devices show very good photovoltaic performance in a wide concentration window (ranging from 0.50 to 1.50 M; Fig. 5b, Table 1 and Supplementary Fig. 18), and even achieve high PCE close to 24% (23.80 ± 0.17%) at very low precursor concentrations (0.5 M), highlighting the remarkable process-resilience of this approach. To the best of our knowledge, this is the highest PCE obtained at such a dilute concentration of the perovskite precursor. The photovoltaic performance achieved across different perovskite compositions with and without co-solvent dilution is summarized in Table 1. We also compared the performance of co-solvent diluted devices to those of state-of-the-art PSCs (PCE > 23%) reported in the literature^14,15,32–37^, as summarized in Fig. 5c. The latter typically employ a 1.5 M concentration to achieve PCE > 23% and even 2.5 M to reach 24% PCE. Encouragingly, our co-solvent dilution strategy allows using less than 80% of the starting perovskite material and toxic solvents to fabricate lab-scale devices with similar high performance, which inspired us to validate our strategy on large-scale module fabrication using industrial spin-coating approaches commonly available in a semiconductor fab.

Based on the exceptional results achieved in small area PSCs prepared with diluted precursors with co-solvent, we realized an up-scaling program through the fabrication of several batches of PSMs with different size substrates and active areas as shown in the inset of Fig. 5d. These modules are formed by 5 cells in series with an active area of 10 cm^2^ and an aperture area of 11 cm^2^ (hereafter nominated as mini modules) and 15 series-connected cells with an active area of 45.6 cm^2^ and an aperture area of 53 cm^2^ (hereafter nominated as large modules). Owing to the applied P1-P2-P3 LASER ablation program we realized mini-modules with a 91% geometric fill factor (GFF) and large modules with 86% and 91% GFF values in order to maximize the ratio of the active area vs. aperture area (Supplementary Fig. 19). Here, with our objective to minimize the use of toxic solvents, we use anisole as a green antisolvent alternative to the typical toxic antisolvents (e.g., chlorobenzene and toluene) for module fabrication^38,39^. The optimized perovskite precursor solution (denoted as target) used for the modules is 100 v% THF diluted FA_0.97_MA_0.03_PbI_2.91_Br_0.09_ solution with a concentration of 0.75 M. Meanwhile, for the fabrication of the large modules, 1.8 ml perovskite precursor solution of control was used for deposition whereas only 1.0 ml of target solution was required, owing to the improved wettability of co-solvent diluted precursor solution on the large area substrates. We attribute this to the decreased contact angle (from 56° to 14°) for promoting improved wettability of the precursor solution on large area substrates (Supplementary Fig. 20).

As shown in Fig. 5e and Supplementary Tables 5 and 6, the target modules show higher efficiency and better device-to-device uniformity (smaller standard deviation); average PCE of 18.14 ± 0.31% and 17.97 ± 0.45% overactive areas of 10 and 45.6 cm^2^, respectively. However, the control modules with the same sizes present an average PCE of 17.14 ± 0.55% and 16.40 ± 0.83% over active areas, respectively. The *I–V* measurement of the champion target large PSMs are shown in Fig. 5f and Supplementary Fig. 21 with photovoltaic parameters summarized in Supplementary Table 7. The champion target large PSM showed a PCE of 18.45% with a steady-state efficiency of 17.15% (maximum power point tracking at AM1.5 G illumination, 65 °C, and 60% RH conditions) over the active area of 45.6 cm^2^ (Supplementary Fig. 21b). The module achieves *V*_oc_, *I*_sc_, and FF of 16.07 V, 69.52 mA, and 75.35%, respectively. The stability of an encapsulated module fabricated by co-solvent dilution strategy has been assessed against thermal stress. As presented in Supplementary Fig. 22, the module shows good stability, maintaining over 90% of initial efficiency after 250 h under thermal stress at 85 °C.

The improved module performance uniformity with co-solvent dilution is attributed to improved wetting and uniformity of the perovskite film properties as compared to the control. This was further investigated by measuring UV–vis absorbance spectra at different positions of large area (10 cm × 10 cm) substrates prepared using target and control solutions (adding the same amount of perovskite solutions on the surface). As shown in Supplementary Fig. 23, co-solvent diluted large-scale perovskite solutions yield more uniform thin film absorbance across the 100 cm^2^ surface area compared to the control perovskite film. The unique characteristics of the THF co-solvent dilution strategy can be well described by Fick’s equation for the nucleation kinetics^40^. Accordingly, achieving a highly supersaturated precursor solution early during spin coating and uniformly across the substrate is a key step for high nucleation density and uniform microstructure across the large substrate surface. Our strategy successfully applied a solvent mixture that effectively wetted the entire substrate and also decreased the amount of solute by utilization of a volatile solvent with less solubility with respect to DMF/DMSO. This combination led to the rapid increase of the concentration of the solute at the particle/solution interface early during spin coating to yield a high viscosity sol which stops the outflow behavior thus pinning the sol film thickness. Upon antisolvent drip, the highly supersaturated sol undergoes nucleation and diffusion-controlled growth. The concentration of the sol accelerates the formation of uniformly sized nuclei during phase transformation. Such an approach represents a new strategy which differs substantially with respect to the published reports about solvent additives (e.g., HX addition^41^, SCN^-^^42^, DIO addition^43^, or other Lewis acid-base adduct formation for slowing down the crystal growth^44^), which can have a similar beneficial effect on the uniformity of the perovskite layer without the downside of leaving residual additives in the perovskite layer thanks to the low interaction strength and fast evaporation rate of THF. Importantly, this demonstrates that module fabrication using industrial spin coating can be achieved without any substantial upscaling efforts in regards to formulation and processing as would normally be required when also translating perovskite films from lab-scale spin coating to alternative manufacturing processes. These results demonstrate that the co-solvent dilution strategy can potentially lead to the commercialization of PSCs and perovskite-Si hybrid tandems fabricated by batch techniques, without the need to switch to completely different coating methods^18–21^, which currently underperform compared to spin-coating.

To demonstrate the economic impact of our co-solvent dilution strategy on large-area perovskite module fabrication cost, we did a cost and waste generation analysis of module fabrication from the co-solvent dilution strategy *versus* traditional high concentrated solutions by industrial spin-coating. As shown in Fig. 5g–i, we calculated the amount of toxic solvent (DMF) and perovskite waste produced, and perovskite material cost for 1 W power generation. The calculation details are included in Supplementary Note 1. Our analysis shows that we save over 1.34 mL of DMF and over 1.51 g of perovskite raw materials and 37.8 $ per W of power generated from the module fabricated with co-solvent dilution strategy compared to the conventional strategy when using industrial spin coating. If we scale these numbers for a solar plant of 1 GW, we can avoid over 1.34 × 10^6^ L of toxic DMF, over 1.51 × 10^6^ kg of toxic perovskite waste, and savings approaching 40 billion $ in terms of material cost compared to conventional inks by spin coating. In this regard, more than 70% of toxic waste, perovskite raw material, and fabrication cost are projected to be reduced for module fabrication with a co-solvent dilution strategy.

### Enhanced precursor solution stability

**Fig. 6:**
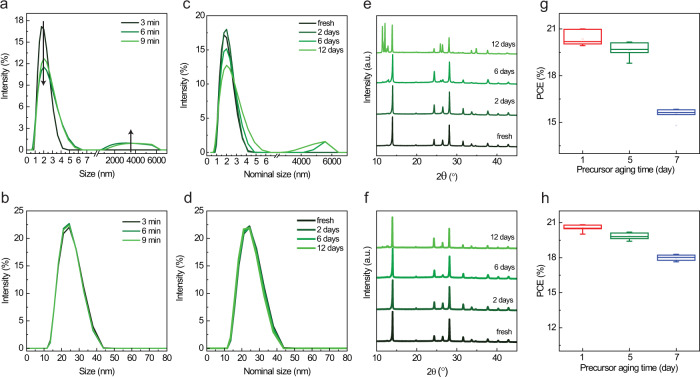
Perovskite precursor stability. **a**, **b** DLS data of fresh precursor solutions **a** without and **b** with co-solvent dilution. The data were collected at different scans (each scan takes 3 min). **c**–**f** DLS data and XRD pattern of aged precursor solutions (**c**, **e**) without and (**d**, **f**) with co-solvent dilution. **g**, **h** Distribution of PCE of PSCs fabricated as a function of the aging time of a precursor solution **g** without and **h** with so-solvent dilution. The perovskite films were used for device fabrication without any surface passivation treatment.

Precursor solution stability is an important issue for reproducible fabrication of PSCs with a wide processing window and for minimizing toxic waste by extending shelf-life as well as for the scalable manufacturing of perovskite modules^20,26,45,46^. Inclusion of some additives has been reported to improve the precursor solution stability^26,47,48^. Encouragingly, we found that our simple co-solvent dilution strategy can significantly extend the shelf-life stability of perovskite precursor solutions without requiring additives. We investigated the stability of various precursor inks by storing them in an inert atmosphere and in the dark. First, we investigated the evolution over time of perovskite precursor colloids of the high-performance double-cation perovskite ((FAPbI_3_)_0.97_(MAPbBr_3_)_0.03_) by dynamic light scattering measurements. As shown in Fig. 6a, a large amount of micron-sized aggregates appeared in the high concentration control precursors, which indicates the perovskite colloids are unstable in the conventional mixtures of DMSO and DMF. In contrast, the co-solvent diluted precursor shows excellent colloidal stability with no change in the size distribution (Fig. 6b). Second, we investigated precursor composition changes with respect to storage time (Fig. 6c, d). To further confirm a link between chemical inhomogeneity of the solution (i.e., formation of large aggregates whose composition is different from the target) and of the prepared film, we checked the XRD of perovskite films made from the control and co-solvent diluted precursors that were aged for different durations. As shown in Fig. 6e, the controlled precursor contained large aggregates after 6 days shows the appearance of impurity phases below 12°. Interestingly, the co-solvent diluted precursor shows superior shelf stability, as the high purity photoactive perovskite phase still formed even after 12 days of aging (Fig. 6f). The UV-Vis absorbance of perovskite film results also confirmed the similar tendency in the XRD results (Supplementary Fig. 24). Importantly, PSCs fabricated with aged precursor solutions exhibit substantially different degradation rates with or without co-solvent dilution (Fig. 6g, h). The devices fabricated from the aged control precursor solutions showed a rapid efficiency drop from 20.35 ± 0.45% (fresh) to 15.54 ± 0.39% (7 days), in agreement with our observation of the film absorbance and crystal structure changes. The devices fabricated from the aged co-solvent diluted precursor solution experience much less efficiency drop from 20.53 ± 0.32% (fresh) to 18.00 ± 0.28% (7 days). The co-solvent dilution strategy can also be employed to enhance the precursor shelf-life of other perovskites (see Supplementary Figs. 25 and 26). Therefore, the processing window of perovskite precursors can be significantly extended by our co-solvent dilution strategy, which also decreases the overall waste of perovskite materials.

In summary, we report a co-solvent dilution strategy for the formulation of stable perovskite precursor inks that yields high efficiency while substantially reducing toxic waste generation during fabrication and through enhanced shelf-life, fabrication cost, widening solar cell processing window, thus taking an important step toward commercialization of small- to medium-scale modules via industrial spin-coating, the most widely used batch solution-processing method in the field. This strategy involves the usage of a volatile co-solvent which dilutes perovskite precursor inks to very low concentrations (<0.5 M) while retaining similar film quality and optoelectronic features as a high concentration (>1.4 M) precursor when using facile spin coating. PCEs of over 24% for a small area PSCs could be achieved with a co-solvent dilution to a level as low as 0.5 M. Furthermore, this co-solvent diluted solution shows good scalability which was demonstrated by fabricating modules with different sizes up to 10 cm × 10 cm. The modules fabricated by co-solvent dilution strategy show higher PCEs and better reproducibility than those of the control modules, making a case for use of spin-coating as a sustainable and low-cost fabrication method for medium-scale manufacturing. To validate the co-solvent dilution mechanism, we employ a combination of experimental and computational methods, including MD calculations as well as advanced in situ characterizations. The co-solvent tuned precursor colloidal properties also contribute to the enhancement of the stability of precursor solution, which extends its processing window thus minimizing the waste. This strategy is also successfully applied across different perovskite compositions, substrate areas, as well as device and module sizes hence making it a universal strategy. Importantly, compared to conventional inks, this strategy reduces more than 70% of toxic waste/solvent, perovskite raw material, and fabrication cost. This work provides a strategy that can potentially accelerate the pace of innovation in perovskite optoelectronics and revolutionize the fabrication of perovskite photovoltaics at a commercial scale.

## Methods

### Materials

Lead iodide (PbI_2_) is purchased from Alfa Aesar. Formamidinium iodide (FAI) and titanium dioxide paste (TiO_2_-30 NRD) are purchased from Greatcell. 2,2′,7,7′-Tetrakis[*N*,*N*-di(4-methoxyphenyl)amino]-9,9′-spirobifluorene (Spiro-OMeTAD) is purchased from Xi’an Polymer Light Technology Corp. Methylammonium lead tribromide (MAPbBr_3_) was purchased from Share Chem. Ultra-dry dimethylformamide (DMF), dimethyl sulfoxide (DMSO), ethanol (EtOH), and chlorobenzene (CB) are purchased from Acros. Cesium iodide (CsI), 4-*tert*-butyl pyridine (TBP), lithium bistrifluorosulfonylimide (LiTFSI), acetonitrile (ACN), tetrahydrofuran (THF), acetylacetone, titanium diisopropoxide bis(acetylacetonate) 75 wt% in isopropanol, and methylammonium chloride (MACl) are purchased from Sigma-Aldrich. Fluorine-doped tin oxide (FTO) (10 Ω/sq) conductive glass is purchased from Nippon Sheet Glass (NSG). (Poly[bis(4-phenyl)(2,4,6-trimethylphenyl)amine) (PTAA: 115 kDa) is purchased from Solaris Chem Inc. All the chemicals are used as received without further purification.

### Perovskite film preparation and laboratory cell fabrication

Fluoride-doped tin oxide glass substrates (FTO, 4.0 mm-thick, 10 Ω/sq, Nippon Sheet Glass) were patterned using zinc powder and concentrated hydrochloric acid (1 M). The patterned FTO substrate (2.5 cm ×1.7 cm) was sequentially cleaned by 2% commercial detergent (Hellmanex) water solution, deionized water, ethanol, and acetone in an ultrasonic bath for 15 min, rinsed with deionized water, and then dried by air blowgun. After O_3_/ultraviolet treatment for 15 min, the 20–40 nm compact layer TiO_2_ (c-TiO_2_) was deposited on cleaned FTO substrate by spray pyrolysis at 450 °C using a precursor solution of titanium diisopropoxide bis(acetylacetonate), 75 wt% in isopropanol is diluted with ethanol with a volume ratio of 1:9 and addition 4% volume ratio of additional acetylacetone and oxygen as carrier gas. After cooling down to room temperature and O_3_/ultraviolet treatment for 15 min, the mesoporous TiO_2_ (mp-TiO_2_) was spin-coated at 4000 rpm for 20 s onto the c-TiO_2_ using a commercial paste (Dyesol 30 NR-D) diluted in ethanol (1:6, weight ratio) to achieve 100–150 nm thickness. After drying at 80 °C for 10 min, the TiO_2_ films were gradually sintered to 450 °C, kept at this temperature for 30 min, and cooled to room temperature. Before use, the films were treated with 0.1 M solution of Li-TFSI in acetonitrile by spin coating at 3000 rpm for 20 s and then were sintered at 450 °C for 30 min. After cooling down to 150 °C, the substrates were transferred to a dry-air glovebox (relative humidity < 15%) for deposition of perovskite films. Perovskite films with different compositions were deposited onto the mp-TiO_2_ substrate from a precursor solution with a different dilute ratio of co-solvent. Considering the solute solubility, a 100–200 v% dilution ratio can be used for most of the perovskite materials. Thanks to the improved wettability, the THF-diluted solution showed very good surface coverage even at a low loading amount, i.e.,10 μL, while a fully covered control perovskite film needs at least 30 μL precursor solution (Supplementary Fig. 27 and Supplementary Videos 1 and 2). Therefore, we use a 30 μL precursor solution for comparison in our study.

1. Typical triple-cation perovskite Cs_0.05_(MA_0.1_FA_0.9_)_0.95_Pb(I_0.9_Br_0.1_)_3_. Briefly, a 30 μL 1.4 M mixed cation Cs_0.05_(MA_0.1_FA_0.9_)_0.95_Pb(I_0.9_Br_0.1_)_3_ perovskite precursor solution with 3 mol% excess of PbI_2_ in the mixed solvent of DMF: DMSO (4/1; v/v) was spin-coated at 2000 rpm (acceleration of 200 rpm s^−1^) and 6000 rpm (acceleration of 2000 rpm s^−1^) for 10 and 30 s, respectively. During the last 10 s of the second spin-coating step, 200 μL of chlorobenzene antisolvent was drop-casted. The perovskite film was then dried on a hot plate at 100 °C for 60 min to form the perovskite layer.
2. High-performance double-cation perovskite (FAPbI_3_)_0.97_(MAPbBr_3_)_0.03_. The double-cation perovskite films were deposited onto the mp-TiO_2_ substrate from a precursor solution containing lead iodide (1.51 M), formamidinium iodide (1.47 M), and methylammonium bromide (0.03 M), lead bromide (0.03 M), and methylammonium chloride (0.6 M) in the mixed solvent of DMF: DMSO (8 /1; v/v). 30 μL precursor solution was spin-coated in a two-step process at 1000 rpm for 10 s and 6000 rpm for 25 s, respectively. During the second step, 200–300 μl of chlorobenzene was dropped on the spinning substrate 5 s prior to the end of the process. The substrates were sequentially heated at 150 °C for 10 min for perovskite crystals formation.
3. Typical single-cation perovskite MAPbI_3_. Briefly, a 30 μL 1.2 M MAPbI_3_ perovskite precursor solution with 3 mol% excess of PbI_2_ in the mixed solvent of DMF: DMSO (4 /1; v/v) was spin-coated at 4000 rpm (acceleration of 2000 rpm s^−1^) for 30 s. During the last 10 s of the second spin-coating step, 200 μL of chlorobenzene antisolvent was drop-casted. The perovskite film was then dried on a hot plate at 65 °C for 10 and 100 °C for 10 min to form the perovskite layer.

For high-performance devices, the perovskite layer was treated with a crown ether complex solution^35^. A hole transport materials solution containing 75 mM spiro-OMeTAD in chlorobenzene with 40 mM Li-TFSI and 270 mM tBP additives was dynamic spin-coated onto the substrate at 3000 rpm for 30 s. The device fabrication is completed with the deposition of the gold electrode (~80 nm) by thermal evaporation.

*PSM fabrication*. In order to fabricate PSMs, we adopted the P1–P2–P3 patterning procedure by means of a Nd:YVO4 ns laser (*λ* = 355 nm). For the large modules, 15 series-connected cells, with cell width of 4.5 and 67.5 mm whereas for the mini-modules 5 series-connected cells, with cell width of 5 mm and 40 mm length with 10 cm^2^ active area were optimized following a systematic procedure and design reported in our previous^49^. A raster scanning laser (Nd:YVO_4_ pulsed at 80 kHz average output power *P* = 10 W) was applied to etch the FTO/glass substrates (Pilkington, 15 Ω cm^−1^). FTO substrates (10 cm × 10 cm for large and 5.6 cm × 5.6 cm for mini modules) were washed with detergent, distilled water, and isopropanol for 5 min for each step, then the P1 process was realized, with a Fluence of 10. 2 J/cm^2^, to insulate the FTO photoanodes of neighboring cells. Next step, the substrates were kept on a hot plate, reaching 460 °C in 40 min. The solution for spray was prepared and deposited as our previous method^39^ reaching a TiO_2_ blocking layer with a thickness of 50 nm. Mesoporous TiO_2_ (Dyesol 30 NR-D) paste was diluted in ethanol (1:6, weight ratio) and deposited by spin coating method at 4000 rpm with an acceleration rate of 2000 for 20 s, then substrates were annealed using the annealing program ramp^39^. 0.034 M fresh solution of Li-TFSI in acetonitrile was prepared^50^ and deposited on the mesoporous layer by spin coating at 2000 rpm for 10 s for large modules and for mini-modules with 3000 for 10 s, and subsequently sintered with the same annealing program of the mesoporous layer. The substrates were immediately transferred to the N_2_-filled glove box while the temperature reached 150 °C before perovskite deposition. The double-cation perovskite solutions were prepared aforementioned as for small-area cells. For the target module, perovskite solution was diluted with THF as a co-solvent with a volume ratio of 1:1. For the large modules, 1.8 mL control and 1.0 mL target perovskite solutions were deposited on the substrate at 3000 rpm for 60 s with an acceleration rate of 1000, respectively; during the last 30 s anisole (a green antisolvent) was spin-cast on the substrate to form the final perovskite following the annealing on the hotplate at 150 °C for 10 min, followed by 100 °C annealing for 10 min. For the mini-modules, perovskite solution was spin-coated in a two-step process at 1000 rpm for 10 s and 5000 rpm for 25 s, respectively. After that, the perovskite layer was treated with a crown ether complex solution^35^. The spiro-OMeTAD solution was prepared according to our reported method^51^. Briefly, 73.2 mg of spiro-OMeTAD in 1 mL CB solvent was prepared, the solution was doped with 26.8 μL of TBP, 16.6 μL of Li-TFSI solution (520 mg in 1 ml of acetonitrile), and 7.2 μL of FK209 cobalt complex (stock solution 375 mg in 1 mL acetonitrile). Then, for the large modules, the prepared spiro-OMeTAD solution was spin-coated with 2000 rpm for 20 s on the perovskite film. For the mini-modules, (Poly[bis(4-phenyl)(2,4,6-trimethylphenyl)amine) PTAA solution was deposited as hole transport material (HTM) following our reported method^39^. Briefly, 0.173 mM solution of 115 kDa PTAA in 1 ml CB was prepared, and the solution was doped with Li-TFSI (1.8 M in acetonitrile), TBP, and FK209-Co(III)-TFSI (0.14 M in acetonitrile) with a ratio of 20:78:2 mol%. After the deposition of HTM layers, the P2 patterning process was applied to remove the ETL-Perovskite-HTLstack from the underneath FTO, to realize vertical contacts between the latter and the subsequently deposited electrode. We operated the P2 step with a Fluence of 163 mJ/cm^2^. Finally, 100 nm gold was deposited via evaporation. After the gold evaporation, the P3 laser patterning step was realized to insulate the counter-electrodes of the single cells, employing a Fluence of 59 mJ/cm^2^.

### Photovoltaic performance measurements

The prepared PSCs were measured using a 300 W Xenon light source from Oriel. The spectral mismatch between AM 1.5 G and the solar simulator was calibrated by a Schott K113 Tempax filter (Prazosopms G; as & Optik GmbH). Before each measurement, the exact light intensity was determined using a calibrated Si reference diode (certified and calibrated by Newport Corporation PV Lab, Bozeman, MT, USA) equipped with an infrared cut-off filter (KG-3, Schott). Keithley 2400 is used for the current–voltage scan by applying an external voltage bias and measuring the response current with a scan rate of 50 mV/s. The device area was 0.25 cm^2^ (0.5 cm × 0.5 cm). The small cells were masked with a black metal mask with an area of 0.16 cm^2^. No preconditioning (e.g., bias and light soaking) was used for the photovoltaic measurement. IPCE was recorded with a commercial apparatus (Aekeo-Ariadne, Cicci Research s.r.l.) based on a 300 W Xenon lamp. The stability of the cells was measured under a white light-emitting diode lamp with biologic MPG2 potentiostat and was performed under the open air. The device area is masked to around 0.13 cm^2^. The spectral mismatch between AM 1.5 G and the solar simulator was calibrated by a Schott K113 Tempax filter, whose light intensity is calibrated with a silicon diode. The light intensity is around 100 mW cm^−2^, and the actual current is adjusted according to the in-time calibration result from the silicon diode. The stability data is acquired from MPP tracking of unencapsulated devices under a continuous nitrogen flow at 25 °C. The stabilized output power of the unencapsulated modules was analyzed through continuous power point tracking under AM1.5 G standard light irradiation, 65 °C temperature, and ~60% R.H. conditions. Thermal stability of the mini-module (PTAA as HTM) was assessed by exposure of the encapsulated modules (previously reported encapsulation procedure^39^) against 85 °C thermal stress (in the oven).

### SEM measurements

The morphologies of the films were characterized using a high-resolution scanning electron microscope (Zeiss Merlin) with an in-lens detector.

### PL, TRPL, and UV–vis measurements

UV–vis absorptions were measured using Varian Cary 500 spectrometer (Varian USA). Photoluminescence lifetime (TCSPC) was measured using an Edinburgh Instruments life spec II fluorescence spectrometer; a picosecond pulsed diode laser (EPL-510, excitation wavelength 510 nm, pulse width <60 ps, fluence < 3 nJ/cm^2^) was used. Photoluminescence spectral photon flux was measured using an Andor Kymera 193i spectrograph and a 660 nm continuous-wave laser set at 1-Sun equivalent photon flux (1.1 µm beam full-width half-maximum, 632 µW); photoluminescence was collected at normal incidence using a 0.1 NA, 110 µm-diameter optical fiber. UV–vis measurement for the large area perovskite films (10 cm × 10 cm) was measured with a BLACK-Comet UV–vis Spectrometer.

### In situ UV–vis transmittance/absorbance and wet film thickness measurements

The in situ UV–vis transmittance measurements were performed using an F20-UVX (Filmetrics) spectrometer inside an N_2_ filled glovebox with moisture and oxygen levels below 0.1 ppm. All the measurements were performed on SnO_2_ coated glass substrates for thinning behavior extraction as TiO_2_ substrates were thick and porous which caused weak and scattered transmittance signals. Transmittance spectra are collected at a repetition rate of 10 Hz and an integration time of 100 ms. Transmittance is converted to absorbance using the relation *A* = 2−log_10_(*T*), where *T* represents the transmittance. The thickness of the wet film is obtained using a model fit of a transparent thin film on glass to individual transmittance spectra (Fig. S13, Supplementary information) using the Filmetrics software, FilMeasure. In this software, the stack of the film is created as air/perovskite film/SnO2/glass/air in transmittance mode. By having initial information on the thickness (*d*) and refractive index (*n*) of glass and SnO_2_ layer as well as the *n* value of the perovskite layer, various *d* values of the wet perovskite layer are given to the model as input until a >99% good of a fit to the spectra is achieved.

### Solvent mass change vs. time

These measurements were conducted inside N_2_ filled glovebox with oxygen and moisture level below 0.1 ppm using a Mettler Toledo XPE105 scale with a glass petri dish placed on it. Plots in Fig. 3b were achieved by running 3 separate measurements in which 20 μl of 200% THF-diluted DMF/DMSO mixture, 20 μl of pure THF, and ~6.67 μl of DMF/DMSO mixture which corresponds to the amount of DMF/DMSO present in 20 μl of 200% THF diluted DMF/DMSO, were dropped onto the glass petri dish. Change in solvent mass due to the evaporation was recorded vs. time.

### Grazing incident wide-angle X-ray scattering

GIWAXS measurements were carried out at CMS beamline, NSLS II. The monochromatic X-ray with the energy of 13.5 keV shone upon the samples at the grazing incident angle of 0.5. A Pilatus800K detector was placed 259 mm away from the sample. The typical exposure time was 10 s.

### Viscosity test

The measurements were performed using a Brookfield DV-1. Viscometer with a small sample adapter (SSA18/13RPY).

### Perovskite film thickness measurements

The measurements were performed using a profilometer (DektakXT, Bruker).

### Solution aging measurements

The solutions were kept in the dark inside an Argon glovebox. The precursors were periodically taken and conducted the relevant measurements.

### Computational methods

We use the Gaussian 16 program package^52^ to perform electronic structure calculations for binding energies. All calculations were carried out at the level of DFT with the Becke-Lee Yang Parr (B3LYP) hybrid functional^53,54^ and with a LanL2DZ^55^ basis set for Pb and 6–311 G + (d, p) basis^55,56^ for C, N, S, O, and H atoms. All Pb-solvent clusters are shown in Supplementary Fig. 9: (a) Pb with 4 DMSO (noted as DMSO in Fig. 2a), (b) Pb with 4 DMF (noted as DMF in Fig. 2a), (c) Pb with 4 THF (noted as THF in Fig. 2a), (d) Pb with 1 THF and 3 DMSO ((noted as DMSO:THF in Fig. 2a)), and (e) Pb with 2 THF, 1 DMF, and 1 DMSO molecules (noted as DMSO:DMF:THF in Fig. 2a). First, we performed geometry optimization of different metal (Pb)–organic (solvent molecules) clusters. To determine the solvent-metal interaction energies, we calculated the energy difference between relaxed configurations (shown in Supplementary Fig. 8), the same solvent configurations with Pb^2+^ removed, and the energy of the isolated Pb^2+^ ion. We performed constant temperature and constant pressure (NPT) Born-Oppenheimer MD (BOMD) simulations of a homogeneous mixture of different precursor solutions. For all simulations we used a typical mixed cations/halides configuration of 7 Pb^2+^, 20 I^−^, 1 Cs^+^, 1 Br^−^, 5FA^+^, 1MA^+^ in 24 solvent molecules. We performed five different precursor simulations of the mixtures of these ions: (a) pure DMF, (b) pure DMSO, (c) pure THF, (d) mixture of THF and DMSO (1:1 mixture), and (e) mixture of THF, DMSO and DMF (3:1:2). All simulations were performed with DFT at the PBE + D3^57,58^ level with double-zeta basis sets (DZVP-MOLOPT for Pb, I, S, O, C, N, H)^59^ and Goedecker–Teter–Hutter (GTH) pseudopotentials^60^ with 560 Ry density cutoff. BOMD simulations were performed with the CP2K package^61,62^. We used a time step of 1 fs and a Nose-Hoover chain thermostat^63^ was used for controlling the temperature at 300 K and the barostat by Martyna et al.^64^ was used for pressure control at 1 atm.

### Reporting summary

Further information on research design is available in the Nature Research Reporting Summary linked to this article.

## Supplementary information

Supplementary Information

Description of Additional Supplementary Files

Supplementary Movie 1

Supplementary Movie 2

Reporting Summary

## Data Availability

Data that support the findings of this study are available in Supplementary Data Files in the Supplementary Information section. Source data are provided with this paper. The simulation data used in this study will be available in the Zendo database under accession code 10.5281/zenodo.5701271. Source data are provided with this paper.

## Source data

Source Data
