# Author Correction: A universal co-solvent dilution strategy enables facile and cost-effective fabrication of perovskite photovoltaics

**DOI:** 10.1038/s41467-022-35132-5

**Published:** 2022-12-13

**Authors:** Hong Zhang, Kasra Darabi, Narges Yaghoobi Nia, Anurag Krishna, Paramvir Ahlawat, Boyu Guo, Masaud Hassan S. Almalki, Tzu-Sen Su, Dan Ren, Viacheslav Bolnykh, Luigi Angelo Castriotta, Mahmoud Zendehdel, Linfeng Pan, Sandy Sanchez Alonso, Ruipeng Li, Shaik M. Zakeeruddin, Anders Hagfeldt, Ursula Rothlisberger, Aldo Di Carlo, Aram Amassian, Michael Grätzel

**Affiliations:** 1 Laboratory of Photonics and Interfaces, École Polytechnique Fédérale de Lausanne, Lausanne, 1015 Switzerland; 2 Department of Materials Science and Engineering, and Organic and Carbon Electronics Laboratories (ORaCEL), North Carolina State University, Raleigh, NC 27695 USA; 3 Centre for Hybrid and Organic Solar Energy (CHOSE), University of Rome Tor Vergata, Rome, 00133 Italy; 4 Laboratory of Photomolecular Science, Institute of Chemical Sciences and Engineering, École Polytechnique Fédérale de Lausanne, Lausanne, 1015 Switzerland; 5 Laboratory of Computational Chemistry and Biochemistry, École Polytechnique Fédérale de Lausanne, Lausanne, 1015 Switzerland; 6 Kimia Solar Research Institute, Kimia Solar Company, Kashan, 87137-45868 Iran; 7 National Synchrotron Light Source II, Brookhaven National Laboratory, Upton, NY 11973 USA; 8 Department of Chemistry – Ångström Laboratory, Uppsala University, 751 20 Uppsala, Sweden; 9 ISM-CNR, Institute of Structure of Matter, National Research Council, via del Fosso del Cavaliere 100, 00133 Rome, Italy

**Keywords:** Materials chemistry, Materials for devices, Solar cells

Correction to: *Nature Communications* 10.1038/s41467-021-27740-4, published online 10 January 2022

This Article contains an error in the spelling of the author Linfeng Pan, which is incorrectly given as Lingfeng Pan. The error has now been corrected in the PDF or HTML versions of the Article.

