# Supplementary material for: A universal co-solvent dilution strategy enables facile and cost-effective fabrication of perovskite photovoltaics
Source: Nat Commun. 2022 Jan 10;13:89. doi: 10.1038/s41467-021-27740-4 (PMC8748698; doi:10.1038/s41467-021-27740-4)
Supplement: Supplementary file 1 — Supplementary Information [file 41467_2021_27740_MOESM1_ESM.pdf]

Supplementary Materials for

A universal co-solvent dilution strategy enables facile and cost-effective fabrication of perovskite photovoltaics

Hong Zhang[1,*,#], Kasra Darabi[2,#], Narges Yaghoobi Nia[3,#], Anurag Krishna[1,4], Paramvir Ahlawat[5], Boyu Guo[2], Masaud Hassan S Almalki[1], Tzu-Sen Su[1], Dan Ren[1], Viacheslav Bolnykh[5], Luigi Angelo Castriotta[3], Mahmoud Zendehdel[3,6], Lingfeng Pan[4], Sandy Sanchez Alonso[1,4], Ruipeng Li[7], Shaik M. Zakeeruddin[1], Anders Hagfeldt[4,8], Ursula Rothlisberger[5], Aldo Di Carlo[3,9,*], Aram Amassian[2,*], Michael Grätzel[1,*]

[1]Laboratory of Photonics and Interfaces, École Polytechnique Fédérale de Lausanne, Lausanne 1015, Switzerland.

[2]Department of Materials Science and Engineering, and Organic and Carbon Electronics Laboratories (ORaCEL), North Carolina State University, Raleigh, North Carolina 27695, United States.

[3]Centre for Hybrid and Organic Solar Energy (CHOSE), University of Rome Tor Vergata, Rome 00133, Italy.

[4]Laboratory of Photomolecular Science, Institute of Chemical Sciences and Engineering, École Polytechnique Fédérale de Lausanne, Lausanne 1015, Switzerland.

[5]Laboratory of Computational Chemistry and Biochemistry, École Polytechnique Fédérale de Lausanne, Lausanne 1015, Switzerland.

[6]Kimia Solar Research Institute, Kimia Solar Company, Kashan 87137-45868, Iran.

[7]National Synchrotron Light Source II, Brookhaven National Laboratory, Upton, New York, 11973, United States.

[8]Department of Chemistry – Ångström Laboratory, Uppsala University, 751 20 Uppsala, Sweden.

[9]ISM-CNR, Institute of Structure of Matter, National Research Council, via del Fosso del Cavaliere 100, 00133 Rome, Italy.

*Correspondence to: hong.zhang@epfl.ch (H.Z.); aldo.dicarlo@uniroma2.it (A.D.C.); aamassi@ncsu.edu (A.A.); michael.graetzel@epfl.ch (M.G.)

# These authors contributed equally.

Supplementary Table 1. The cost of perovskite raw materials.

Material (purity)	Price (USD/gram)[a]
Lead(II) iodide (99.999% trace metals basis, perovskite grade)	24.6
Lead(II) bromide (99.999% trace metals basis)	10.6
Cesium iodide (~10 mesh, 99.999% trace metals basis)	10.0
Methylammonium bromide (⩾99%, anhydrous)	28.2
Formamidinium iodide (⩾99%, anhydrous)	28.2

[a] https://www.sigmaaldrich.com (April 2021)

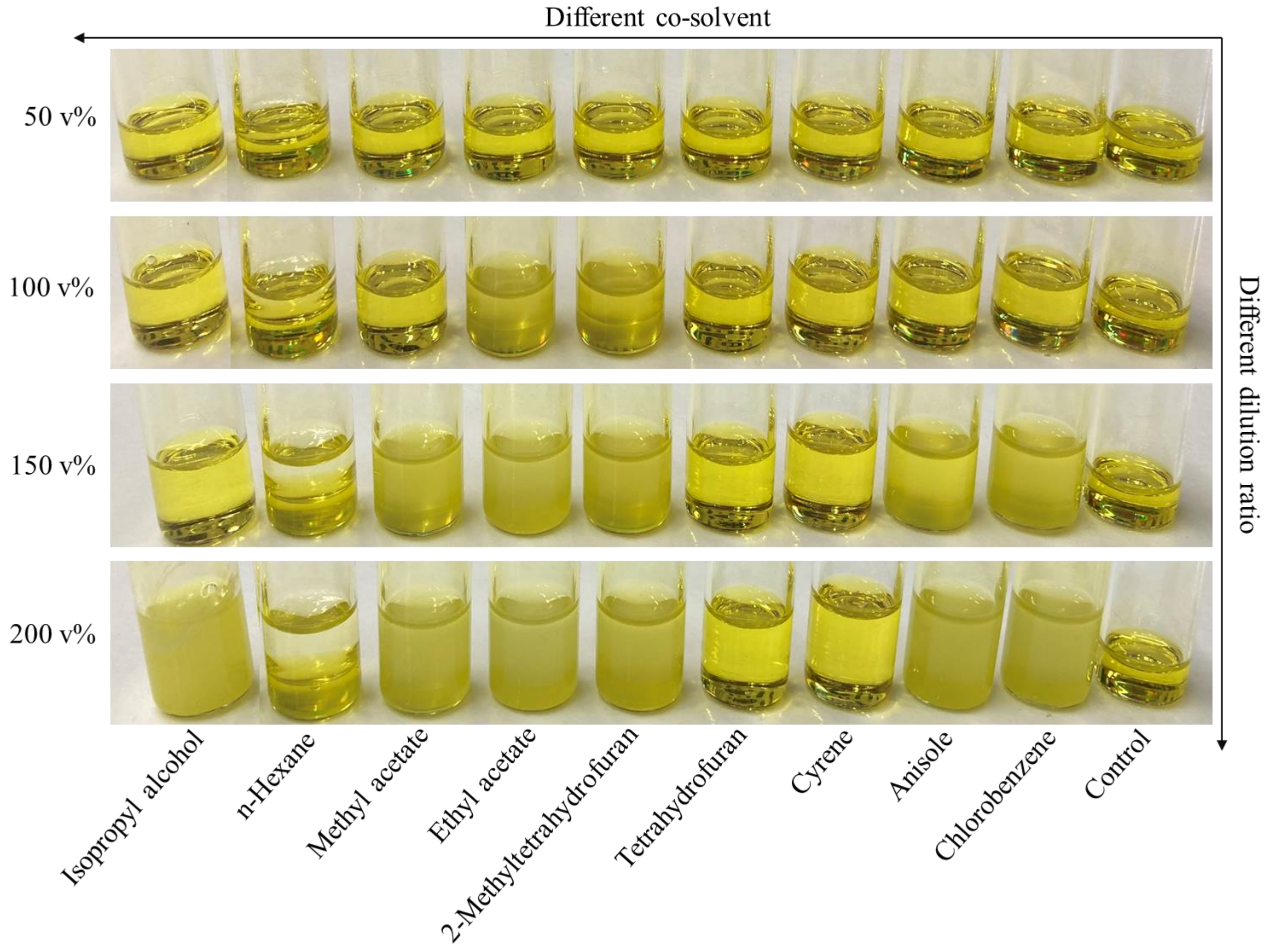

Supplementary Fig. 1. Photographic images of triple-cation perovskite precursor diluted with different co-solvents.

Supplementary Table 2. Screening of the green co-solvent.

Co-solvent	Boiling point (°C)	Dilution ratio	Solubility[a]	Film formation
Isopropyl alcohol (IPA)	82.5	50 v%	√	√
		100 v%	√	√
		150 v%	√	√
		200 v%	×	×
Methyl acetate	57	50 v%	√	√
		100 v%	√	√
		150 v%	×	×
		200 v%	×	×
Ethyl acetate	77	50 v%	√	√
		100 v%	×	×
		150 v%	×	×
		200 v%	×	×
Cyrene	227	50 v%	√	×
		100 v%	√	×
		150 v%	√	×
		200 v%	√	×
Tetrahydrofuran (THF)	66	50 v%	√	√
		100 v%	√	√
		150 v%	√	√
		200 v%	√	√
2-Methyltetrahydrofuran	80.2	50 v%	√	√
		100 v%	×	×
		150 v%	×	×
		200 v%	×	×
Hexane	69	50 v%	×	×
		100 v%	×	×
		150 v%	×	×
		200 v%	×	×
Anisole	153.8	50 v%	√	√
		100 v%	√	√
		150 v%	×	×
		200 v%	×	×
Chlorobenzene		50 v%	√	√
		100 v%	√	√
		150 v%	×	×
		200 v%	×	×

[a] √ refers to soluble or good film quality; × refers to insoluble or poor film quality.

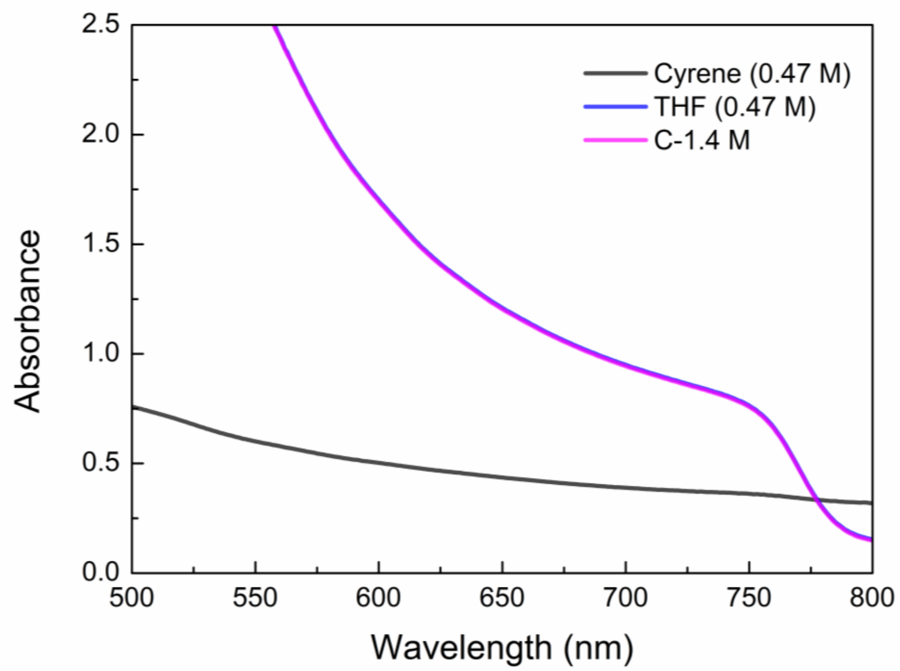

Supplementary Fig. 2. UV-Vis absorption spectra of triple-cation perovskite film prepared from different co-solvent diluted (200 v% Cyrene and 200 v% THF) precursors and high concentration control precursor.

Although Cyrene diluted precursor shows very good solubility, the prepared perovskite film quality is very poor.

Supplementary Table 2. Solvent properties used in this work.

Physical properties	DMF	DMSO	THF
Boiling point [°C] at 1 atm	154	189	66
Vapor pressure [kPa] at 20 °C	0.5200	0.0556	17.5986
Human toxicity (10^{-6} DALYS/ kg emitted)[1]	4.06	0.01	0.06
Energy required for drying with air velocity of 4 m s^{-1} (kJ kg^{-1})[1]	5247.8	6123.4	0.8
Gutmann's donor number (D_N)[2]	26.6	29.8	20

http://www.stenutz.eu/chem/solv21.php

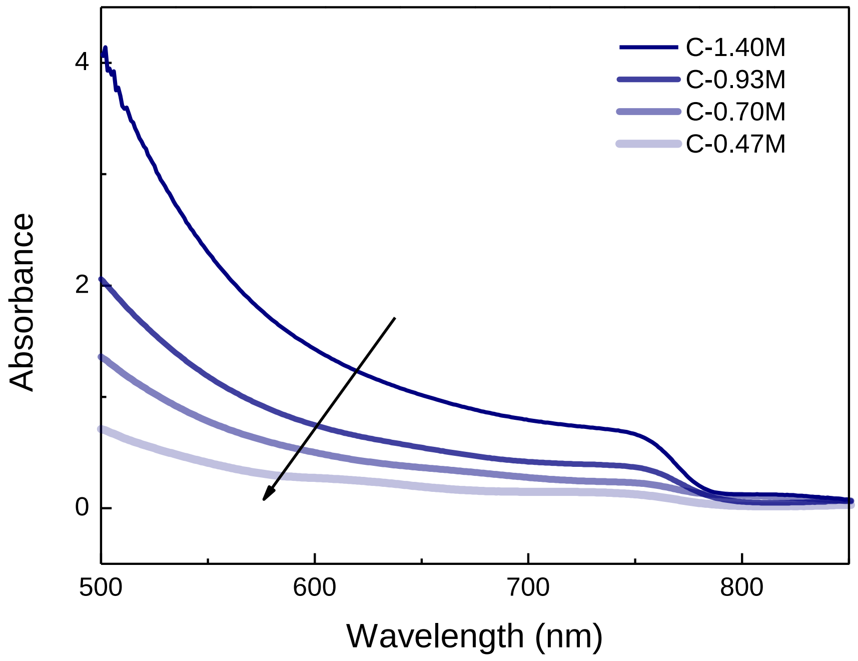

Supplementary Fig. 3. UV-Vis absorption spectra of triple-cation perovskite film prepared from different concentration of precursor.

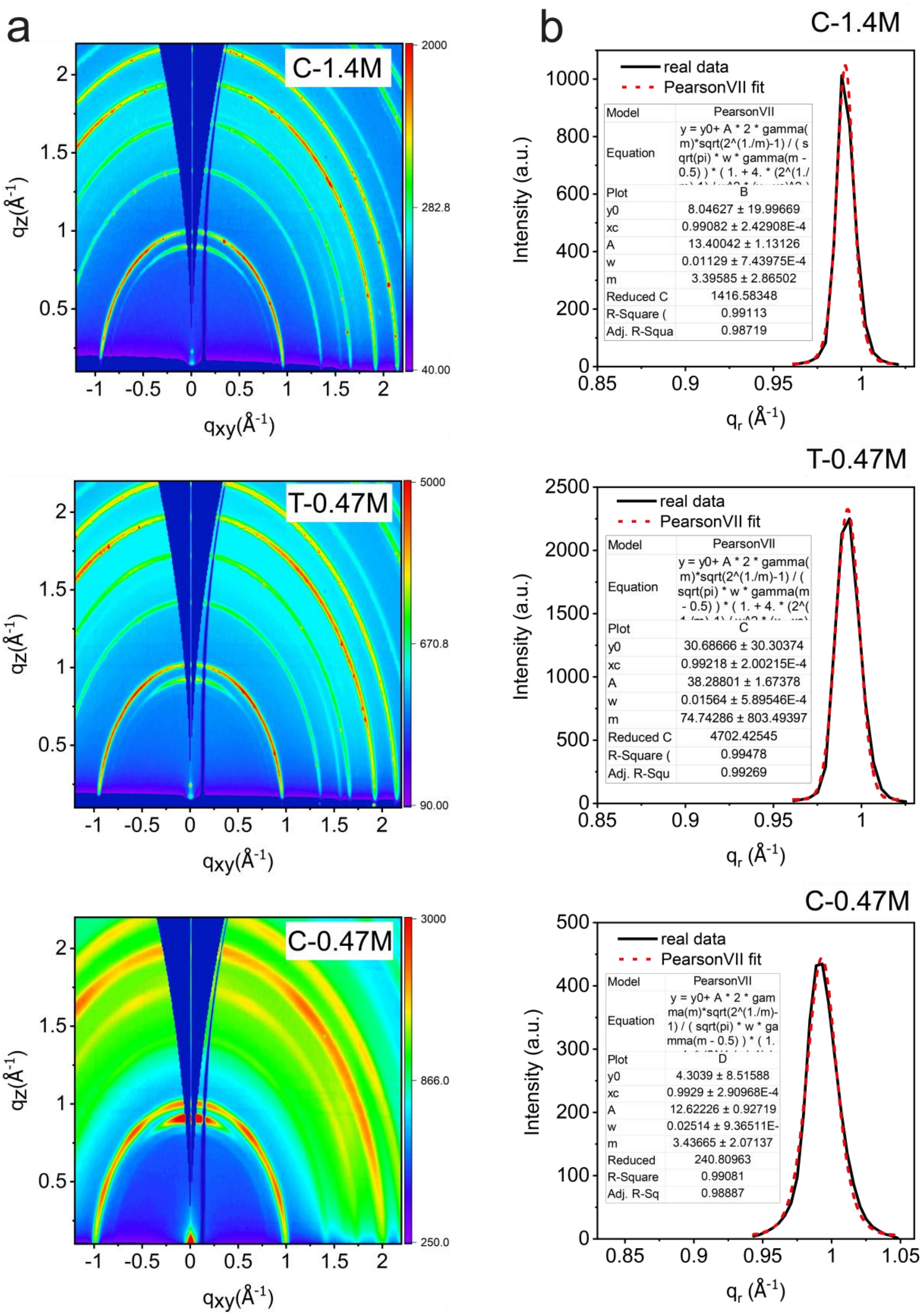

Supplementary Fig. 4. (a) GIWAXS results of the control, THF-diluted and DMF/DMSO diluted samples. (b) GIWAXS peak fitting of the perovskite phase.

Supplementary Table 3. Peak fitting FWHM of the GIWAXS data Supplementary Fig. 5.

$$y = y_0 + A\frac{2\Gamma(m)\sqrt{2^{\frac{1}{m}} - 1}}{\sqrt{\pi}\Gamma\left(m - \frac{1}{2}\right)w}\left[1 + 4\frac{2^{\frac{1}{m}}}{w^2}(x - x_c)^2\right]^{-m}$$	
**Sample name**	**FWHM (GIWAXS - $q = 1$ Å^{-1})**
C-1.4 M	0.0113 ± 0.0007
T-0.47 M	0.0156 ± 0.0006
C-0.47 M	0.0251 ± 0.0009

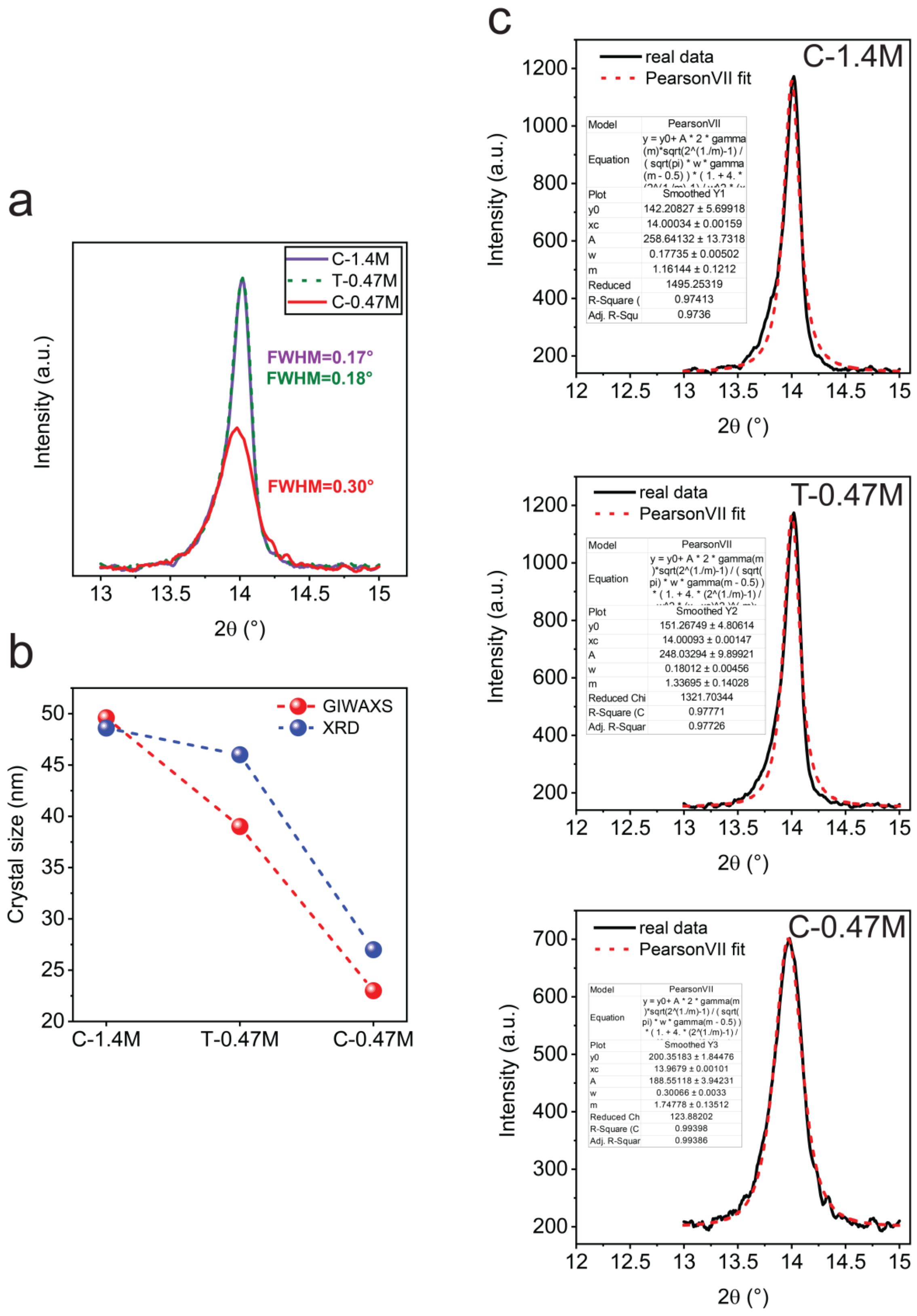

Supplementary Fig. 5. (a) XRD results of the control, THF-diluted and DMF/DMSO diluted samples. (b) Change of crystal size versus composition extracted by quantitative analysis of perovskite peak in XRD and GIWAXS. (c) XRD peak fitting of the perovskite phase.

Supplementary Table 4. Peak fitting FWHM of the XRD data in Supplementary Fig. 6.

$$y = y_0 + A\frac{2\Gamma(m)\sqrt{2^{\frac{1}{m}} - 1}}{\sqrt{\pi}\Gamma\left(m - \frac{1}{2}\right)w}[1 + 4\frac{2^{\frac{1}{m}}}{w^2}(x - x_c)^2]^{-m}$$	
**Sample name**	**FWHM (XRD - $2\theta = 14^{\circ}$)**
C-1.4 M	0.177 ± 0.005
T-0.47 M	0.180 ± 0.005
C-0.47 M	0.301 ± 0.003

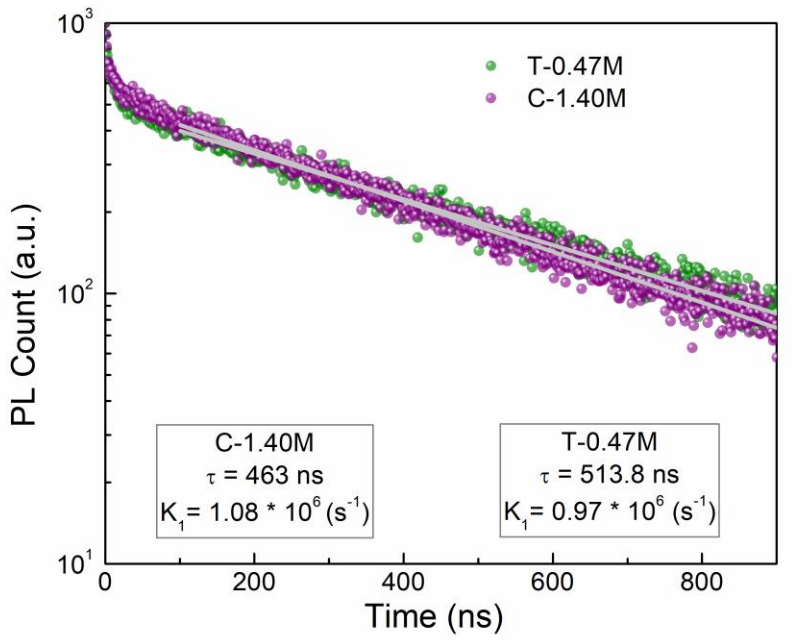

Supplementary Fig. 6. TRPL spectra of triple-cation perovskite film prepared from control (C-1.40M) and 200 v% THF diluted precursor (T-0.47M).

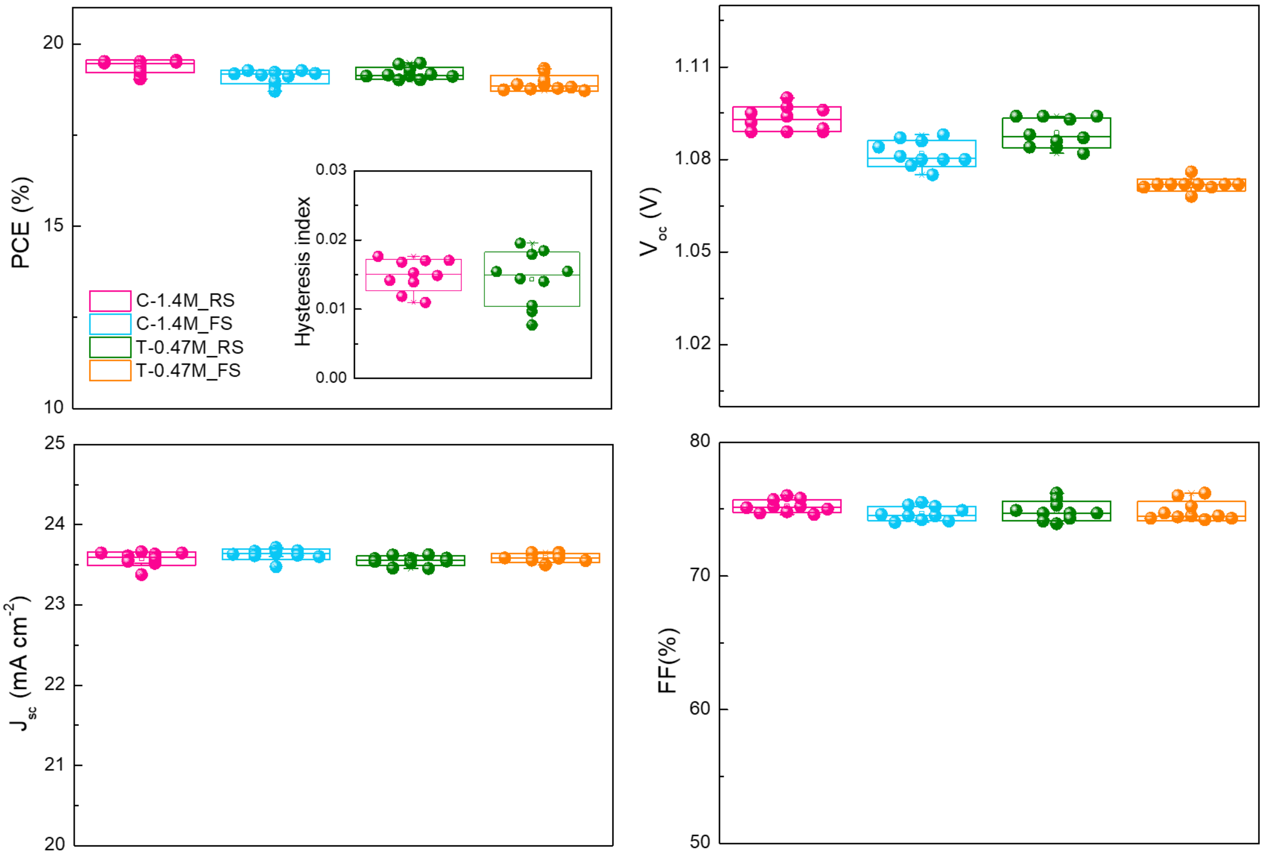

Supplementary Fig. 7. Summary of photovoltaic metrics for triple-cation perovskite-based PSCs without (control) and with (target) 200 v% THF dilution. FS = Forward scan; RS = Reverse scan.

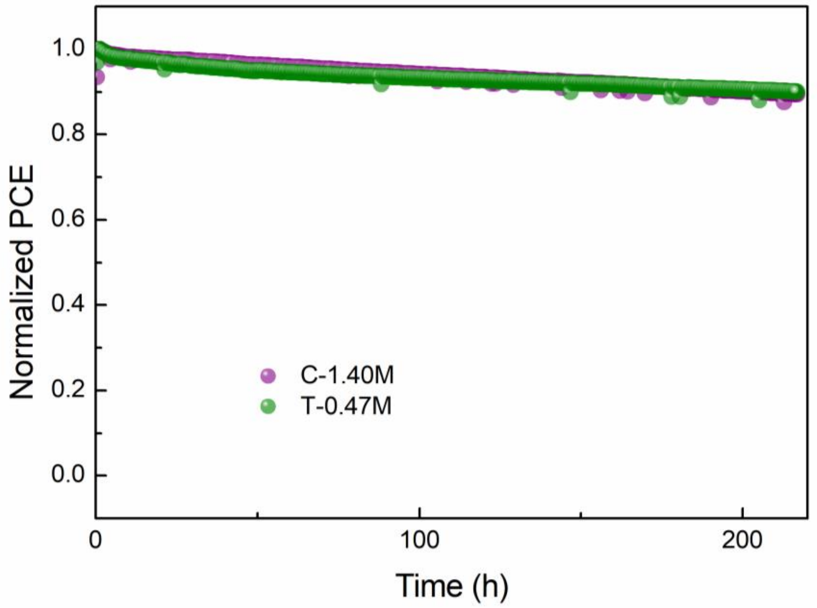

Supplementary Fig. 8. Operational stability was evaluated by maximum power point tracking measured with the unencapsulated device under full solar illumination (AM 1.5 G, 100 mW/cm^2 in N_2, 25 °C).

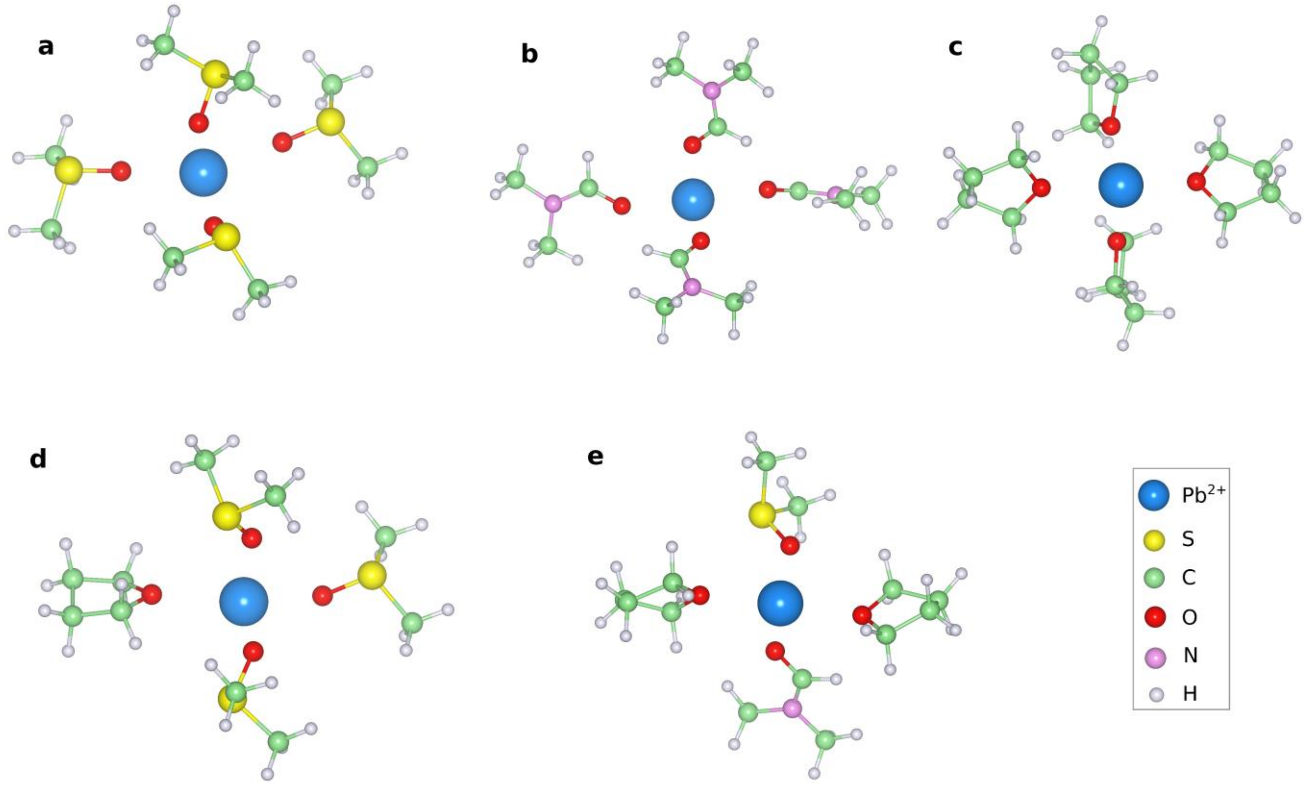

Supplementary Fig. 9. Prototypical tetracoordinated Pb^{2+} clusters with different solvents: (a) DMSO, (b) DMF, (c) THF, (d) mixtures of DMSO and THF, and (e) mixtures of DMF, DMSO and THF. All the atoms are labeled with their element symbols. All species are shown with balls and sticks representations.

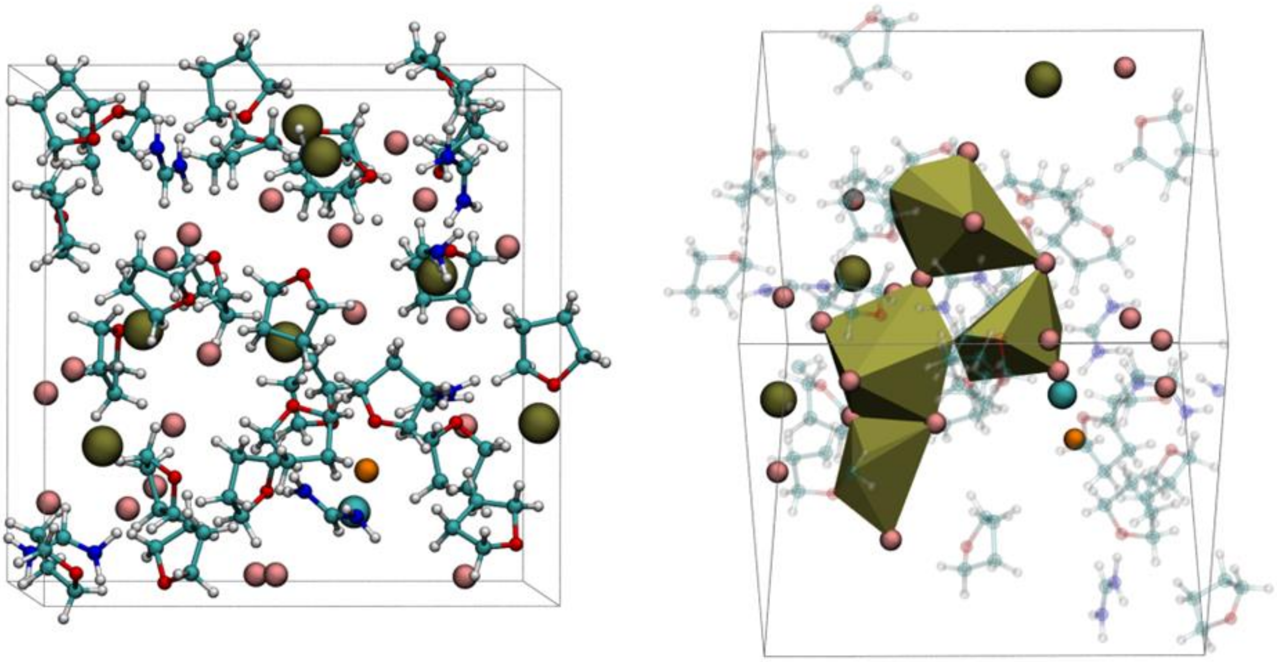

Supplementary Fig. 10. Snapshots from ab initio MD simulations of perovskite precursor solutions in pure THF. Left panel shows the initial configuration and right panel shows the final configuration after ~15 ps. All species are shown in balls and sticks representations. Pb ions are shown with golden color, I with pink color, Cs with greenish blue, Br with orange, C with light blue, N with dark blue,O with red, S with yellow and H atoms with white color. In the right panel, Pb coordination polyhedra are shown within a 3.6A cutoff. In the right panel, solvent molecules are shown in transparent to better guide the eyes.

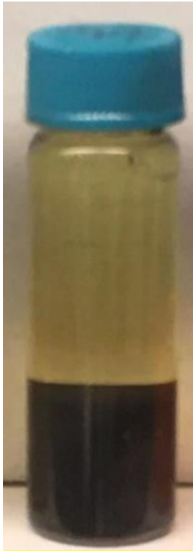

Supplementary Fig. 11. Photograph images of triple-cation perovskite materials in pure THF.

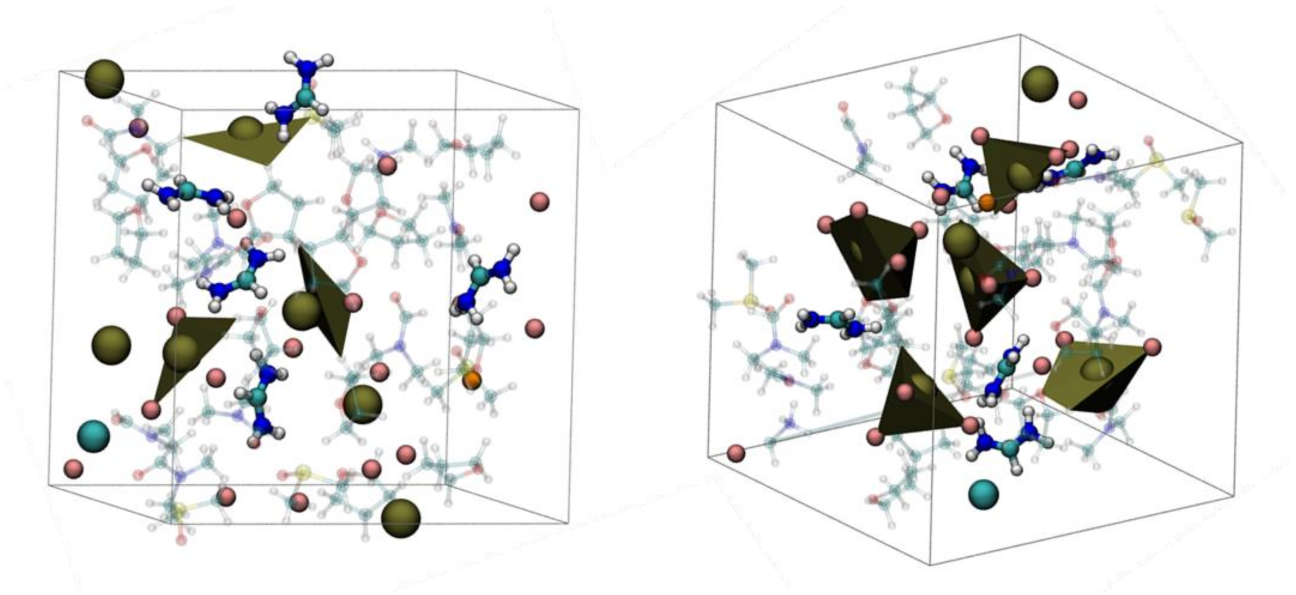

Supplementary Fig. 12. Snapshots from ab initio MD simulations of perovskites precursor solutions in DMSO/DMF/THF mixture. Left panel shows the initial configuration and right panel shows the final configuration after ~15 ps. All species are shown in balls and sticks representations. Pb ions are shown with golden color, I with pink color, Cs with greenish blue, Br with orange, C with light blue, N with dark blue,O with red, S with yellow and H atoms with white color. In the right panel, Pb coordination polyhedra are shown within a 3.6A cutoff. In the right panel, solvent molecules are shown in transparent to better guide the eyes.

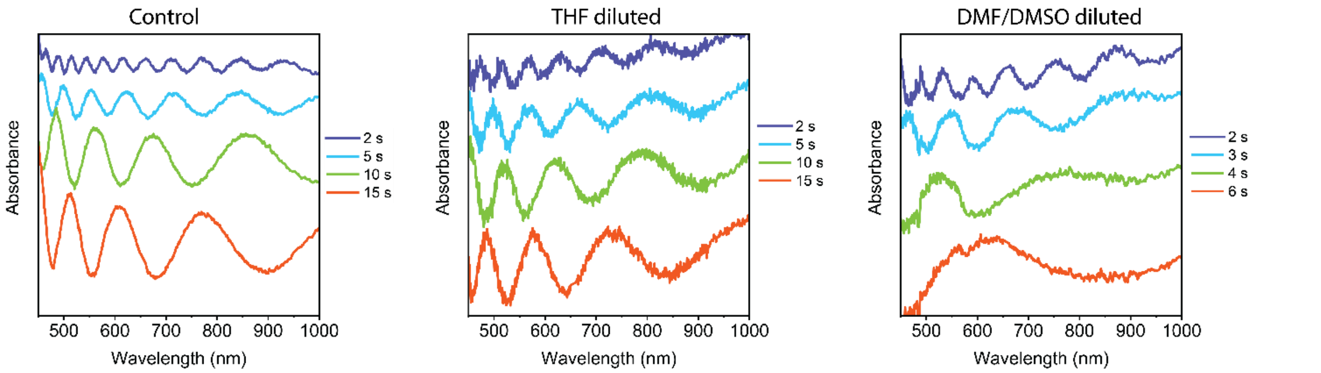

Supplementary Fig. 13. Oscillating fringes of three different solutions during spin coating.

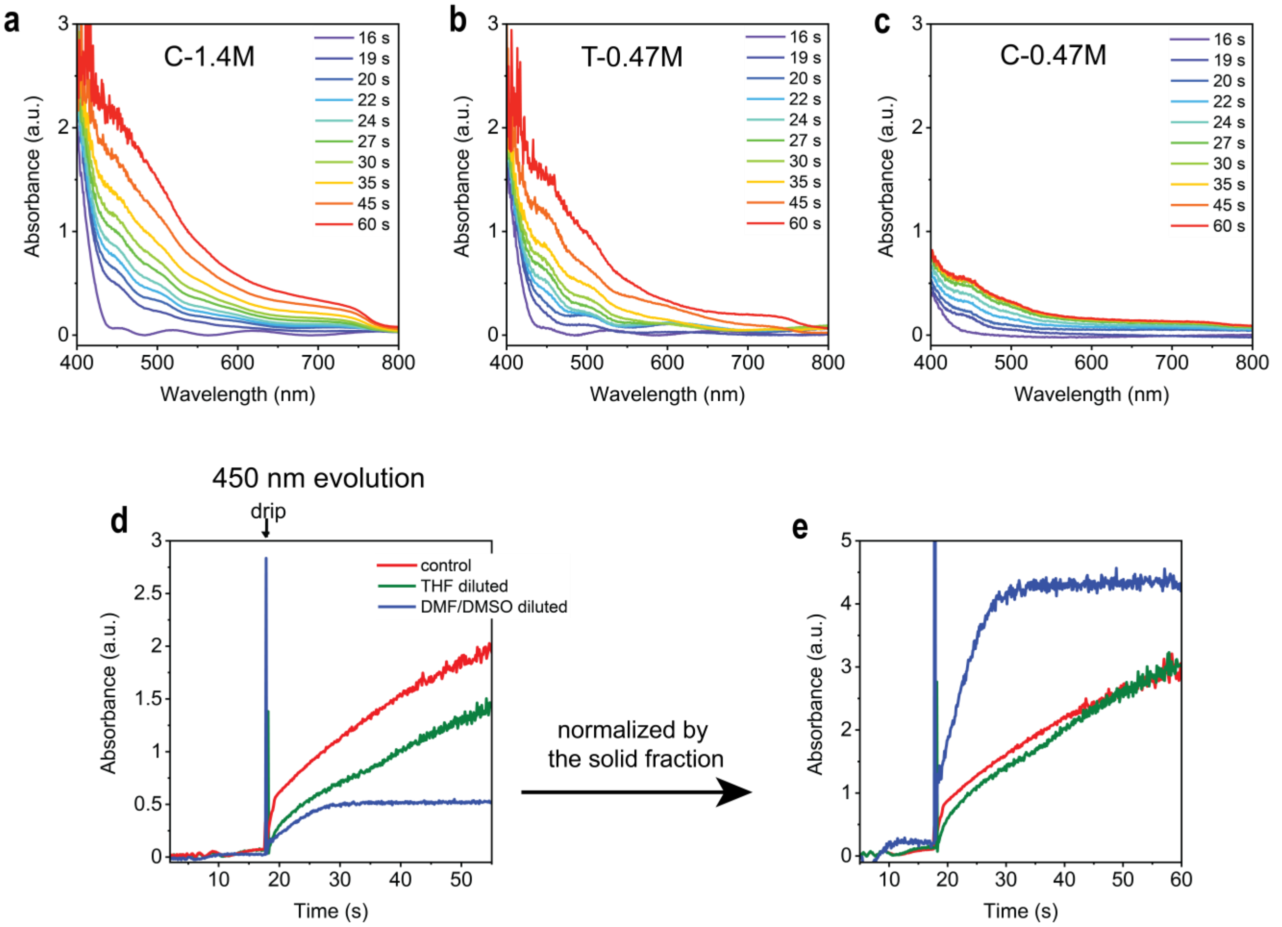

Supplementary Fig. 14. (a-c) *In-situ* UV-Vis absorbance spectra during spin coating at different times for C-1.4M, T-0.47M, and C-0.47M solutions. (d) The evolution of 450 nm wavelength versus time. (e) normalized absorbance of 450 nm evolution.

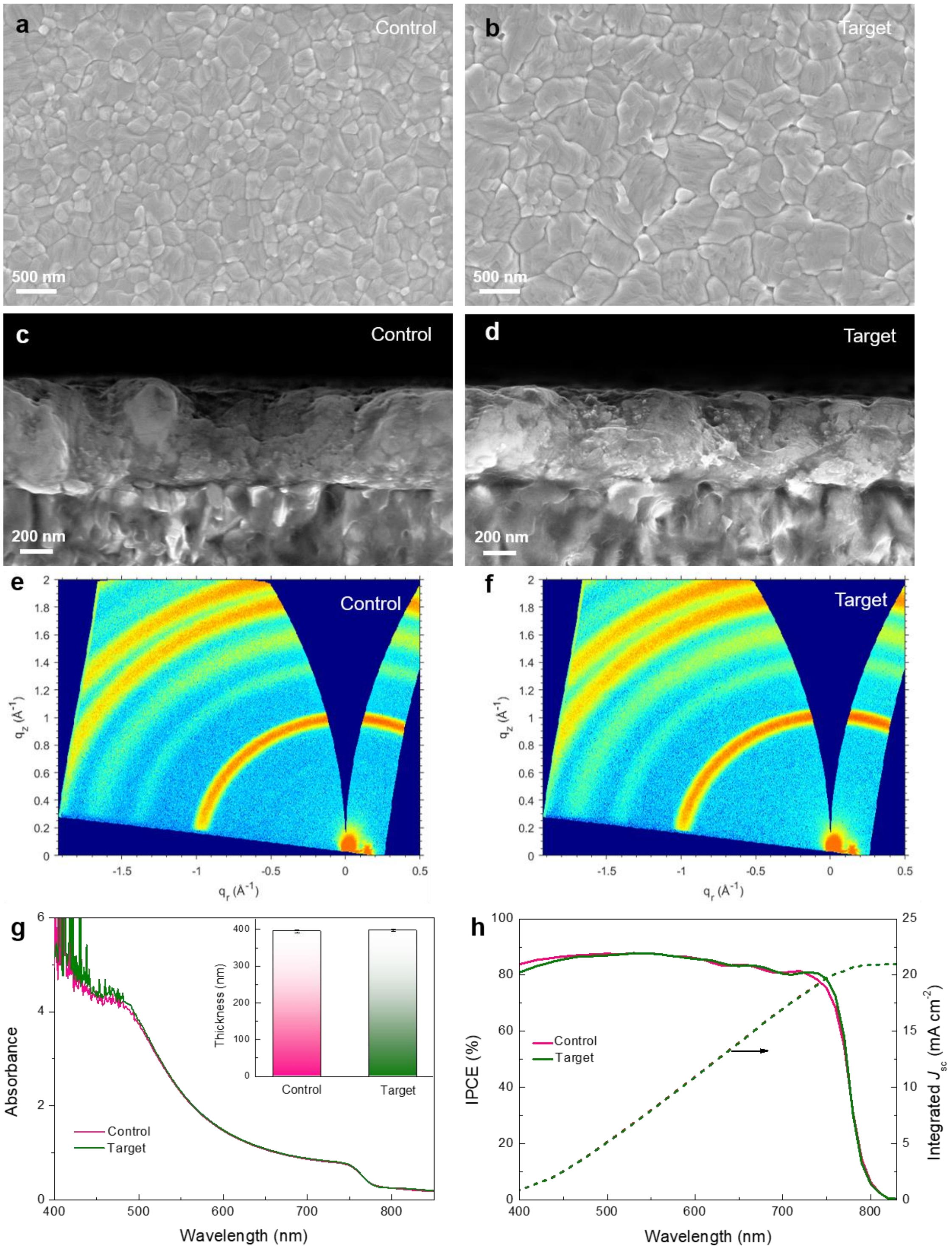

Supplementary Fig. 15. (a-d) SEM images, (e, f) GIWAXS images, and (g) UV-vis spectra of $MAPbI_3$ perovskite films prepared from the precursor solution without (control) and with (target) 200 V% THF dilution. The insert of (g) shows the thickness of the perovskite films. (h) The corresponding IPCE spectra of $MAPbI_3$-based PSCs as depicted in Fig. 4a.

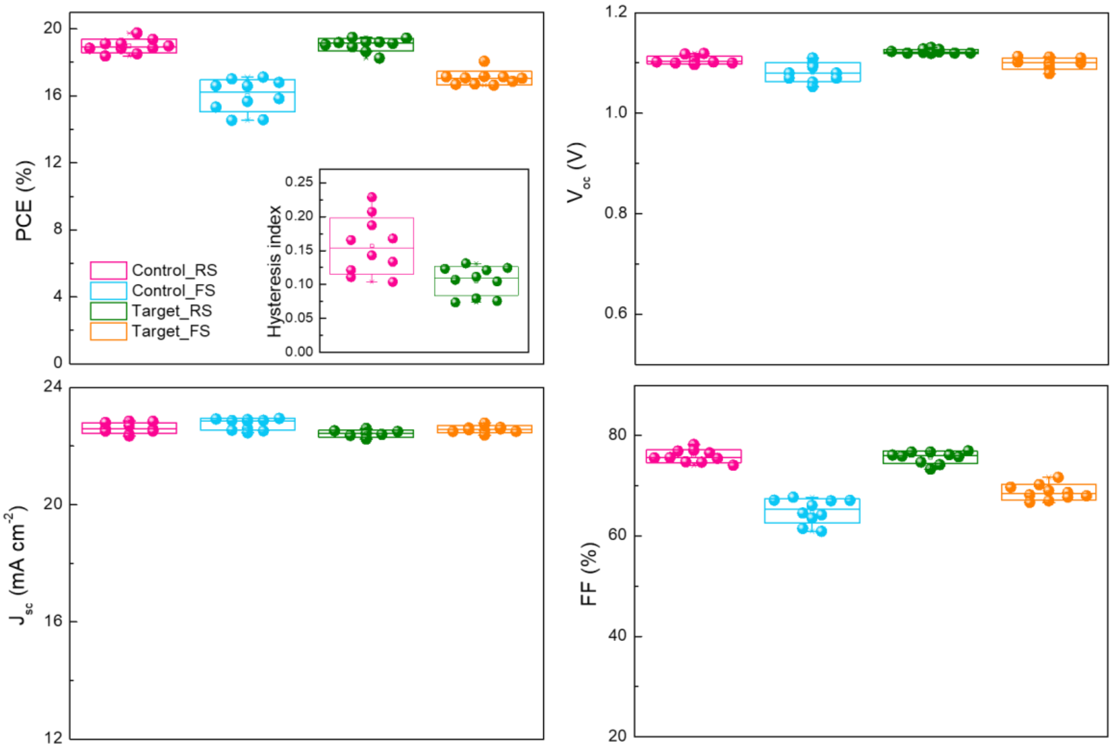

Supplementary Fig. 16. Summary of photovoltaic metrics for $MAPbI_3$-based PSCs without (control) and with (target) 200 v% THF dilution.

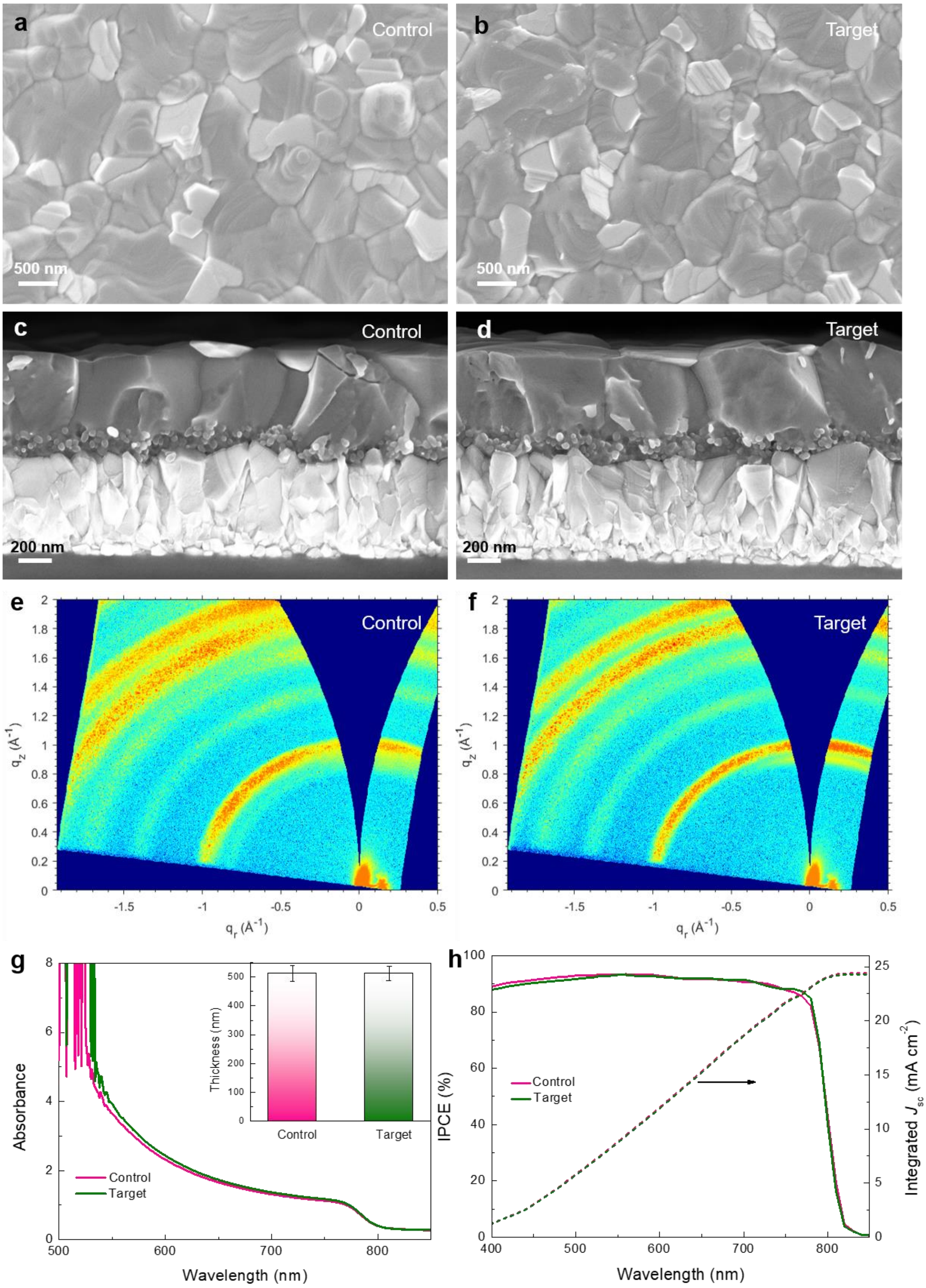

Supplementary Fig. 17. (a-d) SEM images, (e, f) GIWAXS images, and (g) UV-vis spectra of double-cation $FA_{0.97}MA_{0.03}PbI_{2.91}Br_{0.09}$ perovskite films prepared from the precursor solution without (control) and with (target) 200 V% THF dilution. The insert of (g) shows the thickness of the perovskite films. (h) The corresponding IPCE spectra of the champion PSCs as shown in Fig. 4b.

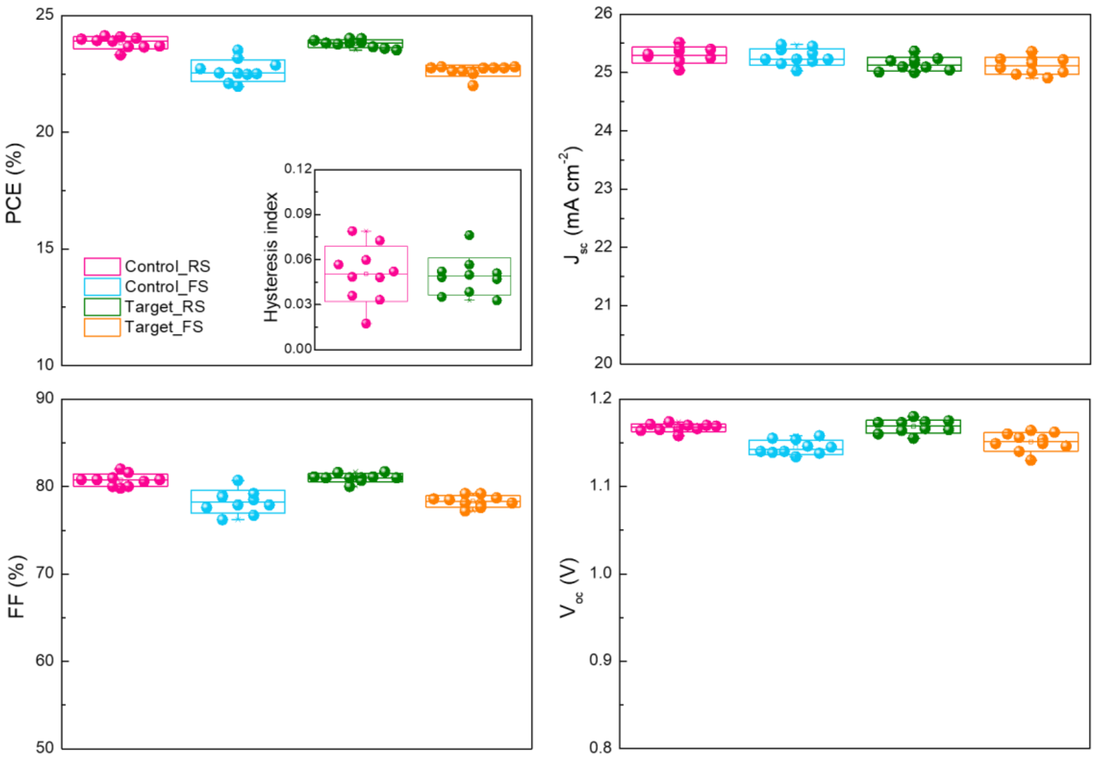

Supplementary Fig. 18. Summary of photovoltaic metrics for $FA_{0.97}MA_{0.03}PbI_{2.91}Br_{0.09}$-based PSCs without (control) and with (target) 200 v% THF dilution.

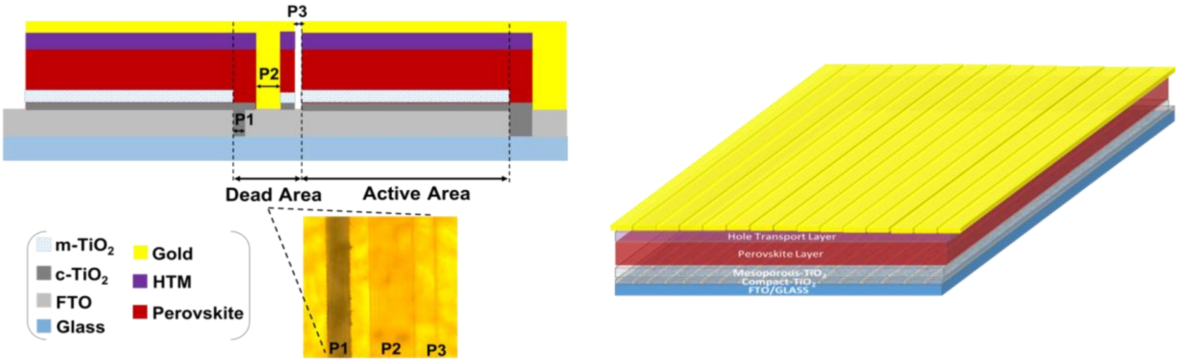

Supplementary Fig. 19. The schematic and microscopic image illustrating the P1/P2/P3 laser programing and geometrical condition of active area and dead area in the fabricated modules.

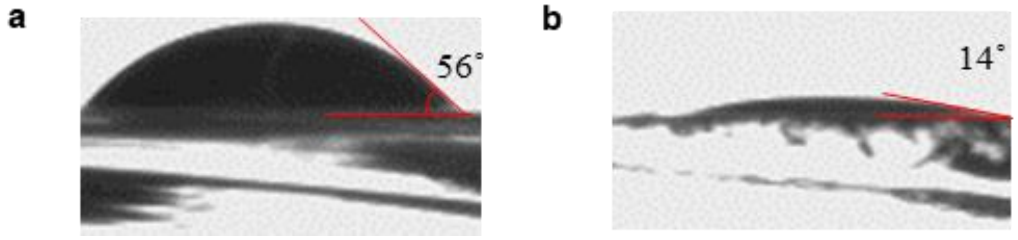

Supplementary Fig. 20. Contact angle of the (a) control and (b) 200 v% THF diluted perovskite precursor solution.

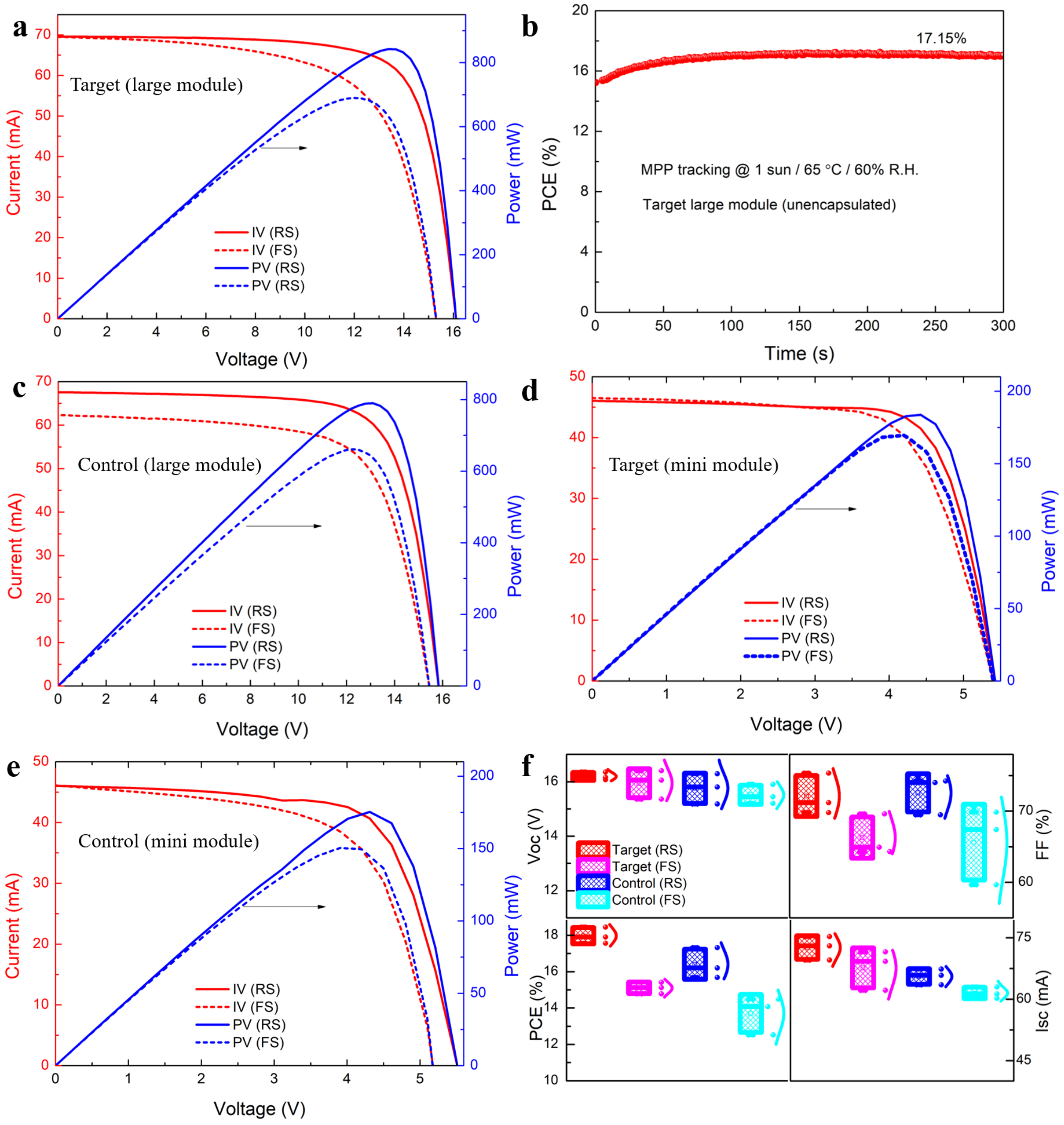

Supplementary Fig. 21. (a) IV-PV curves and of the champion large module fabricated via THF dilution in forward and reverse scan of the voltage (300 mV/s scan rate). (b) PCE tracking at maximum power point of a large perovskite module in (a). (c) IV-PV curves of the best control large module in forward and reverse scan of the voltage (300 mV/s scan rate). IV-PV curves of the best mini-modules in forward and reverse scan of voltage (200 mV/s scan rate) for THF diluted (d) and control (e) mini-modules. Statistical comparison of the photovoltaic parameters including PCE (f), V_{oc} (g), I_{sc} (h) and FF (i) of the fabricated large modules.

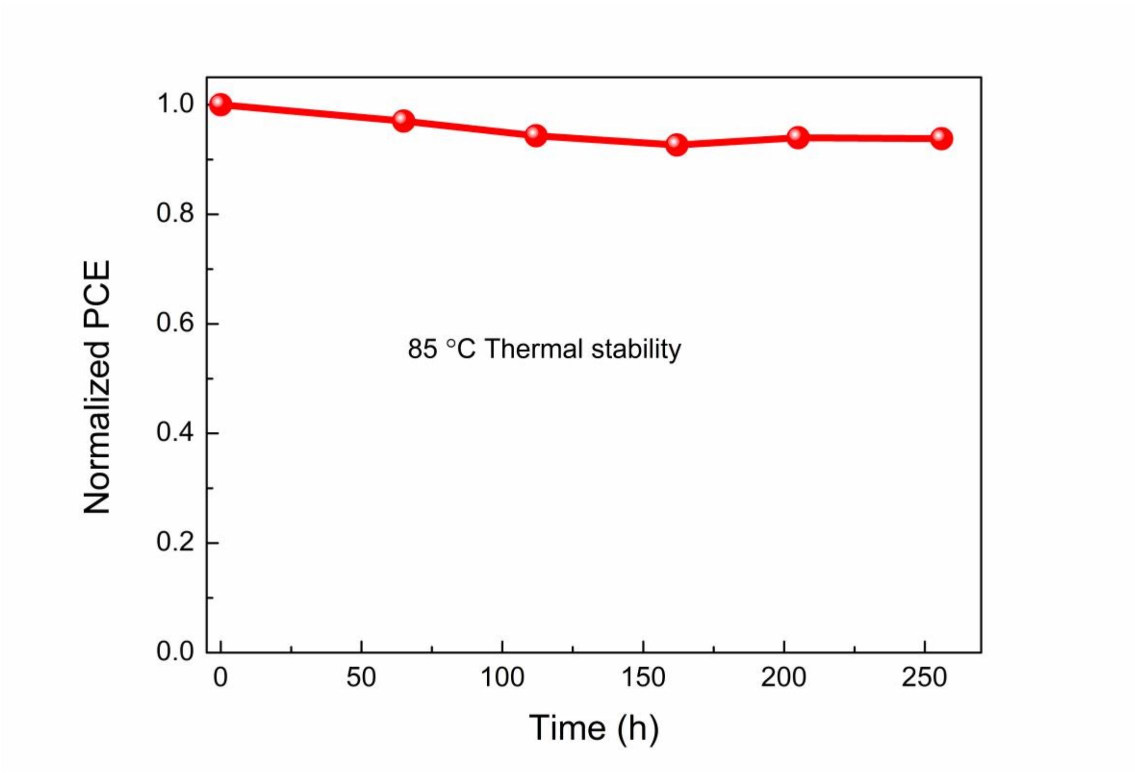

Supplementary Fig. 22. Thermal stability assessment of an encapsulated perovskite solar module fabricated by co-solvent dilution strategy.

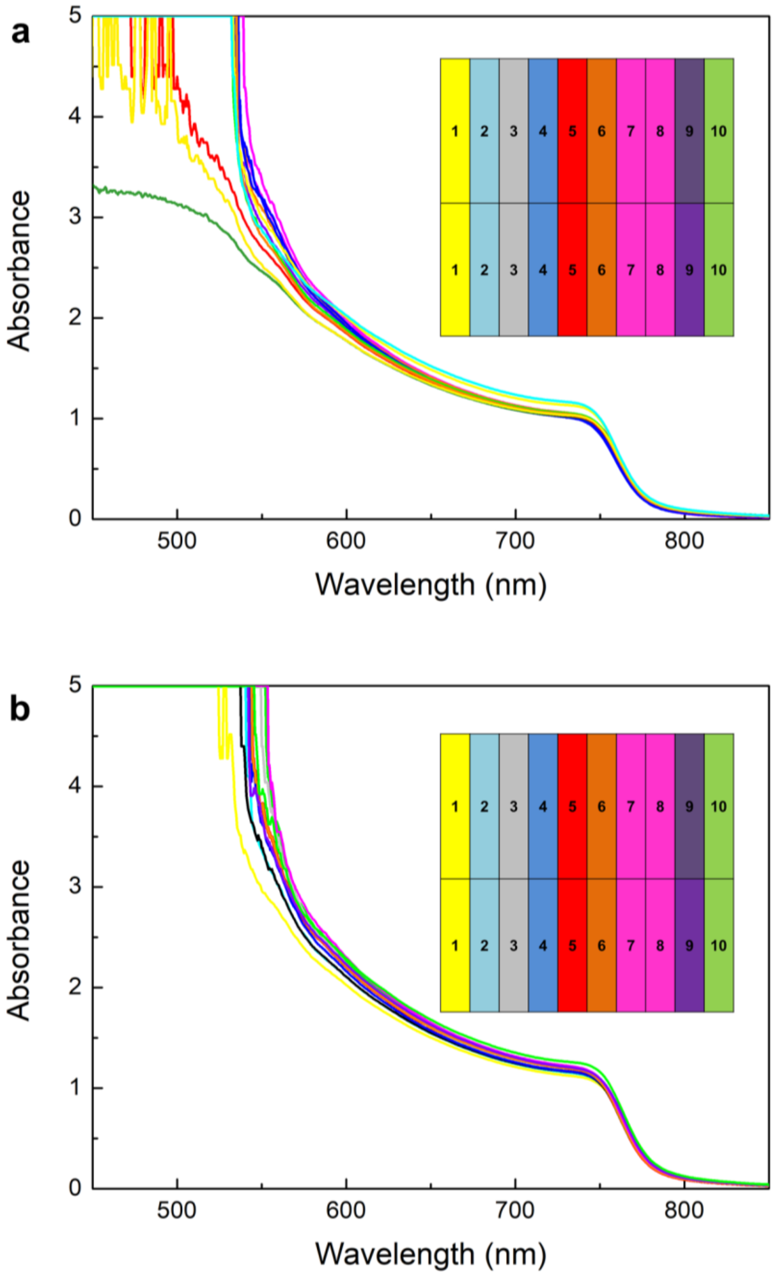

Supplementary Fig. 23. UV-vis absorption spectra of large-area (10 cm × 10 cm) perovskite film prepared from (a) control (C-1.5M) and (b) 100v% THF diluted precursor solution (T-0.75M).

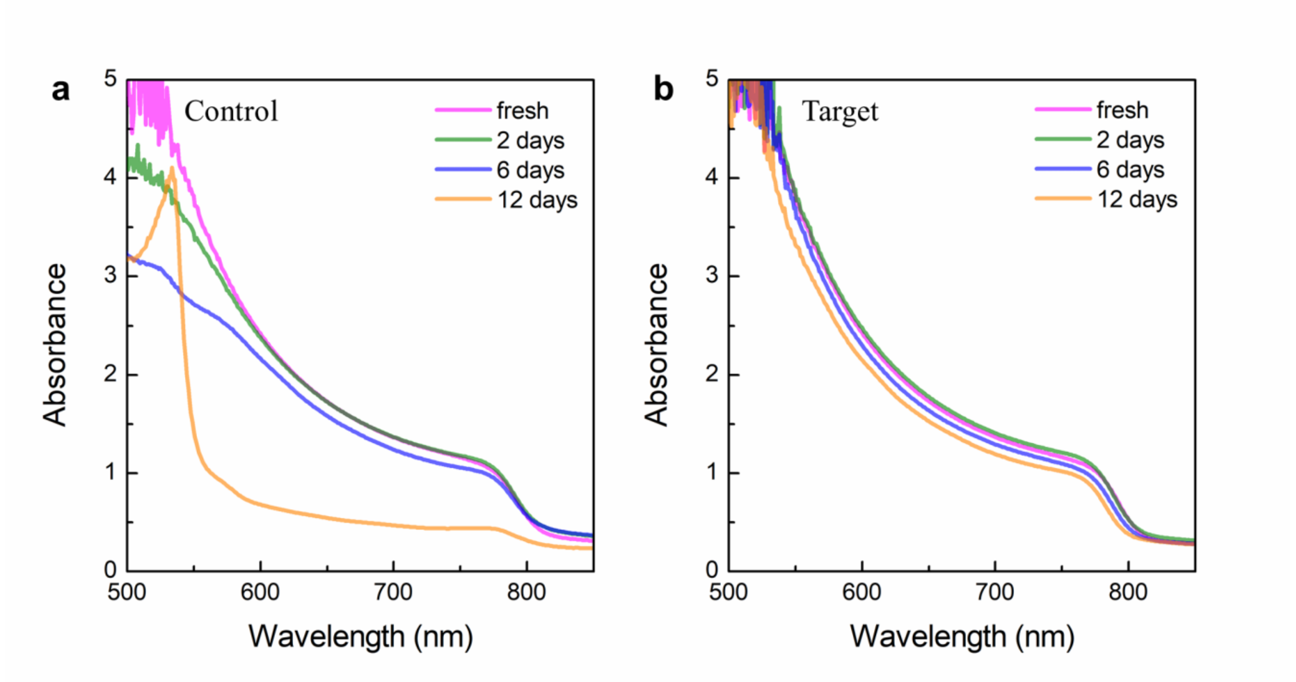

Supplementary Fig. 24. UV-Vis absorptance of aged $FA_{0.97}MA_{0.03}PbI_{2.91}Br_{0.03}$ perovskite precursor solutions (a) without (C-1.5M) and (b) with co-solvent dilution (T-0.5M).

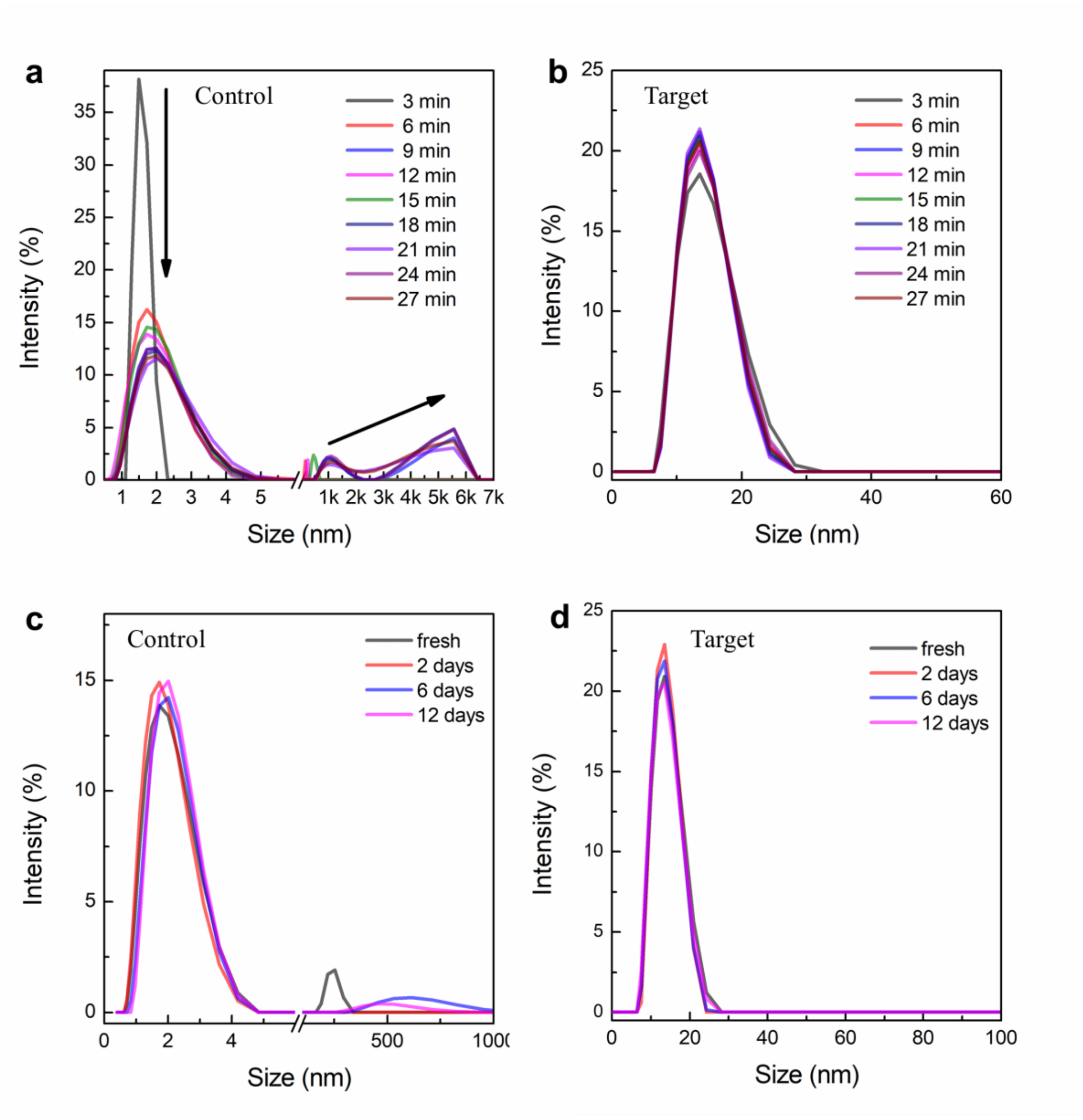

Supplementary Fig. 25. DLS data of fresh $Cs_{0.05}(MA_{0.1}FA_{0.9})_{0.95}Pb(I_{0.9}Br_{0.1})_3$ triple-cation perovskite precursor solutions (a) without (C-1.4M) and (b) with co-solvent dilution (T-0.47M).

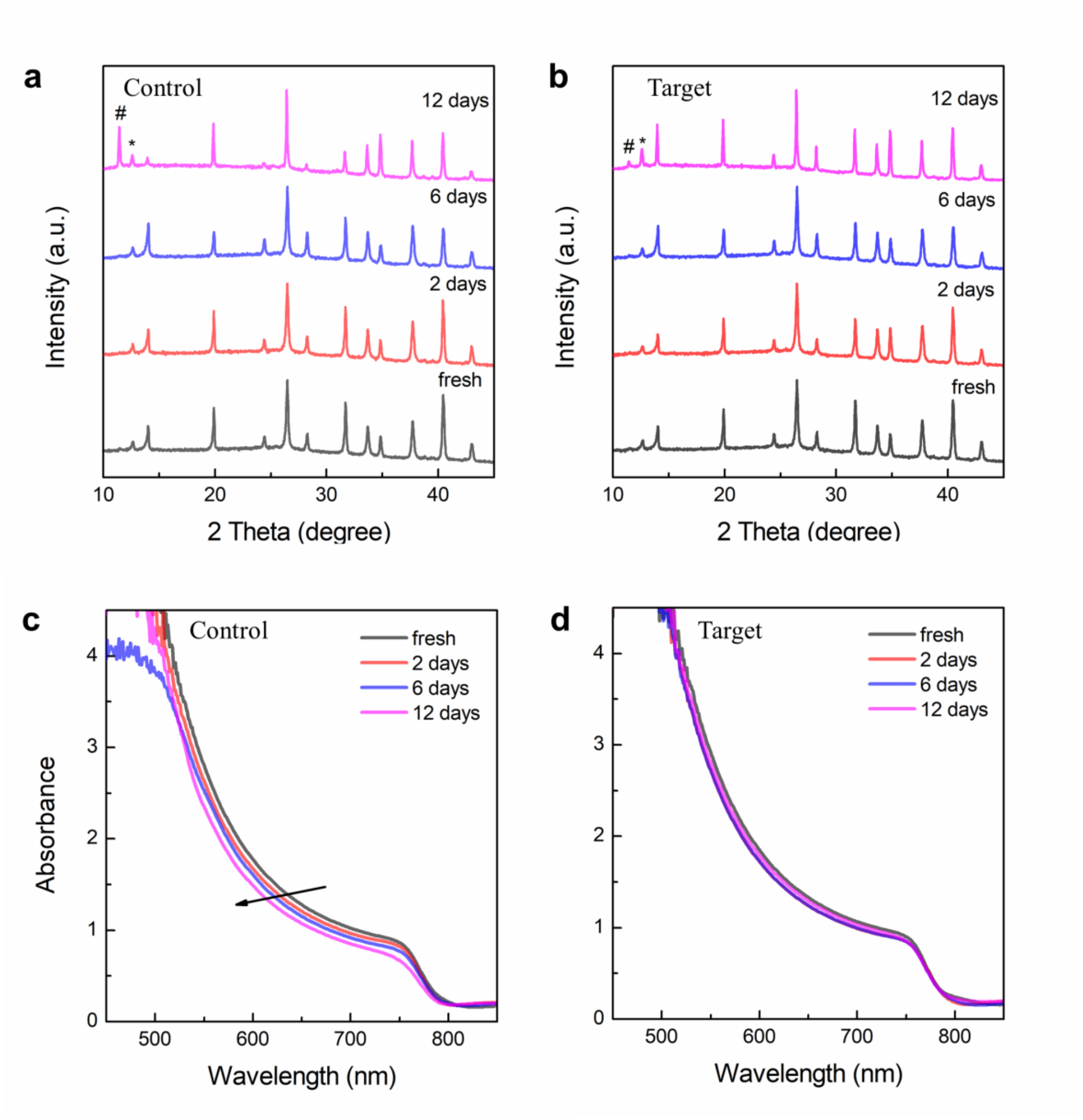

Supplementary Fig. 26. XRD pattern and UV-Vis absorptance of aged triple-cation perovskite $Cs_{0.05}(MA_{0.1}FA_{0.9})_{0.95}Pb(I_{0.9}Br_{0.1})_3$ precursor solutions (a, c) without (C-1.40M) and (b, d) with co-solvent dilution (T-0.47M).

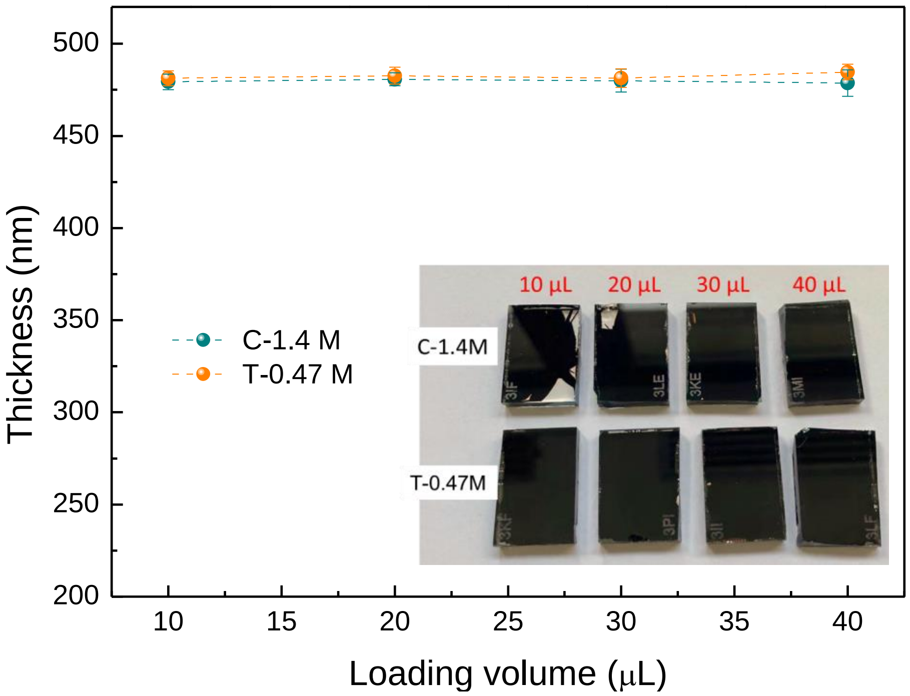

Supplementary Fig. 27. Dependance of thickness and substrate coverage of perovskite film on the loading volume of the solution. Triple-cation perovskite and a FTO/c-TiO2 substrate with a size of 2.5 cm x 1.7 cm were employed for this study.

Supplementary Table 5. Photovoltaic performances of perovskite solar modules with different geometrical fill factor and different perovskite inks.

Module	Active area (cm^2)	Aperture area (cm^2)	Geometrical FF(%)	V_{oc} (V)	I_{sc} (mA)	FF (%)	P_{max} (mW)	PCE (%) on aperture area	PCE (%) on active area	AverageP CE (%)
	45.6	53.0	86	16.07	69.52	75.35	841.96	15.90	18.45	
THF diluted	48.0	52.5	91	16.35	75.26	69.80	859.01	16.30	17.90	17.97 ±0.45
	47.8	52.5	91	16.16	72.99	71.20	839.84	16.00	17.57	
	45.6	53.0	86	15.80	67.31	74.24	789.57	14.90	17.32	
Control	45.6	53.0	86	15.18	65.82	74.01	739.57	13.94	16.21	16.40 ±0.83
	45.8	52.5	87	16.29	63.50	69.49	718.83	13.64	15.68	

Supplementary Table 6. Photovoltaic performances of perovskite solar modules (10 cm^2 active area) and 91% geometrical fill factor fabricated by different perovskite inks.

Module (10 cm^2 active area)	Sweep	V_{oc} (V)	Isc (mA)	FF (%)	PCE (%) on aperture area	PCE (%) on active area
THF diluted (Best)	Reverse	5.45 ±0.03 (5.43)	45.30 ±0.08 (45.36)	73.56 ±1.51 (74.63)	16.51 ±0.28 (16.71)	18.14 ±0.31 (18.36)
	Forward	5.25 ±0.22 (5.41)	44.35 ±1.97 (45.75)	69.89 ±1.86 (68.58)	14.80 ±0.89 (15.43)	16.27 ±0.98 (16.96)
Control (Best)	Reverse	5.40 ±0.16 (5.51)	43.33 ±2.12 (44.82)	73.37 ±3.41 70.96)	15.59 ±0.50 (15.95)	17.14 ±0.55 (17.53)
	Forward	5.14 ±0.30 (5.35)	46.62 ±0.02 (46.63)	61.78 ±2.04 (60.34)	13.45 ±0.34 (13.69)	14.78 ±0.37 (15.04)

Supplementary Table 7. Photovoltaic performances of the champion large module in forward and reverse scan of the voltage (300 mV/s scan rate) in Supplementary Fig. 19a.

Module (45.6 cm^2 active area)	Scan direction	V_{oc}(V)	I_{sc}(mA)	FF (%)	P_{max}(mW)	PCE (%) on active area
THF diluted (Best)	Reverse	16.07	69.52	73.35	841.96	18.45
	Forward	15.35	69.21	64.91	689.88	15.12
Control (Best)	Reverse	15.80	67.31	74.24	789.57	17.32
	Forward	15.44	61.29	69.85	661.15	14.49

Supplementary Note 1. Cost and Waste analysis

Here we use double-cation double halide $(FAPbI_3)_{0.97}(MAPbBr_3)_{0.03}$ perovskite for cost and waste analysis. We assume the PCE of 10 cm x 10 cm module (active area 50 cm^2) is 20%, which delivers 1W under one-sun (100 mW/cm^2) illumination. Since the wettability of perovskite precursor is significantly improved, 2 mL control and 1.5 mL target precursor solution are used for module fabrication. The cost and waste calculation are depicted in detail as follow.

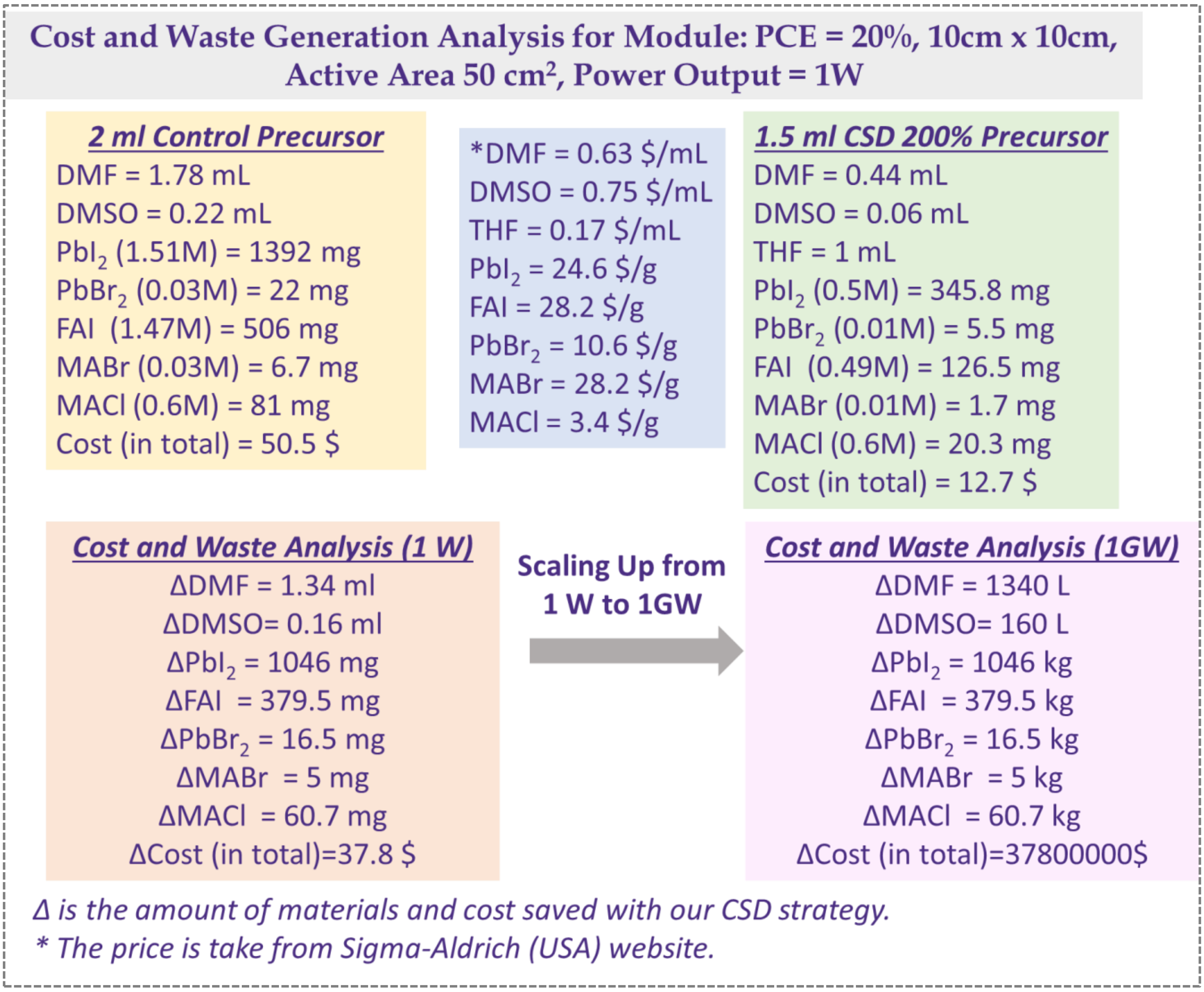

Supplementary References

1. R. Vidal *et al.*, *Nat. Sustain.* **4**, 277–285(2021).
2. J. C. H. Jr. *et al.*, *ACS Energy Lett.* **3**, 92−97 (2018).
