# Supplementary material for: A universal co-solvent dilution strategy enables facile and cost-effective fabrication of perovskite photovoltaics
Source: Nat Commun. 2022 Jan 10;13:89. doi: 10.1038/s41467-021-27740-4 (PMC8748698; doi:10.1038/s41467-021-27740-4)
Supplement: Supplementary file 2 — Description of Additional Supplementary Files [file 41467_2021_27740_MOESM2_ESM.pdf]

Title: Supplementary Movie 1.
Description: The wetting behavior of control (C-1.4 M) triple-cation perovskite solution (30 µL solution was dropped on a FTO/c-TiO2 substrate with a size of 2.5 cm x 1.7 cm).

Title: Supplementary Movie 2.
Description: The wetting behavior of THF-diluted (T0.47 M) triple-cation perovskite solution (30 µL solution was dropped on a FTO/c-TiO2 substrate with a size of 2.5 cm x 1.7 cm).
