# Supplementary material for: A universal co-solvent dilution strategy enables facile and cost-effective fabrication of perovskite photovoltaics
Source: Nat Commun. 2022 Jan 10;13:89. doi: 10.1038/s41467-021-27740-4 (PMC8748698; doi:10.1038/s41467-021-27740-4)
Supplement: Supplementary file 5 — Reporting Summary [file 41467_2021_27740_MOESM5_ESM.pdf]

nature research

Corresponding author(s): Michael Grätzel; Hong Zhang; Aram Amassian; Aldo Di Carlo

Solar Cells Reporting Summary

Nature Research wishes to improve the reproducibility of the work that we publish. This form is intended for publication with all accepted papers reporting the characterization of photovoltaic devices and provides structure for consistency and transparency in reporting. Some list items might not apply to an individual manuscript, but all fields must be completed for clarity.

For further information on Nature Research policies, including our data availability policy, see Authors & Referees.

ü Experimental design

Please check: are the following details reported in the manuscript?

1. Dimensions

Area of the tested solar cells

- [x] Yes
- [ ] No

The device area was 0.25 cm2 (0.5cm x 0.5 cm) and the module area was 100 cm2 (10cm x 10 cm), as described in the Materials and Methods .

Explain why this information is not reported/not relevant.

Method used to determine the device area

- [x] Yes
- [ ] No

Described in Methods and Materials (Photovoltaic performance measurements). A black metal mask with an area of 0.16 cm2 (0.4cm x 0.4 cm) and 53 cm2 are used to define the active area of small cell and module, respectively.

Explain why this information is not reported/not relevant.

2. Current-voltage characterization

Current density-voltage (J-V) plots in both forward and backward direction

- [x] Yes
- [ ] No

The manuscript provides current density - voltage (JV) plots in both forward and backward direction (Fig. 2f, Fig. 5a,b, Fig. S21).

Not applicable since the highlight of this work are new concept of perovskite inks formulation.

Voltage scan conditions
For instance: scan direction, speed, dwell times

- [x] Yes
- [ ] No

Voltage scan conditions are reported in the manuscript and described in the Methods and Materials (Photovoltaic performance measurements).

Explain why this information is not reported/not relevant.

Test environment
For instance: characterization temperature, in air or in glove box

- [x] Yes
- [ ] No

The current-voltage characteristics are obtained under ambient temperature and air conditions, as reported in the manuscript.

Explain why this information is not reported/not relevant.

Protocol for preconditioning of the device before its characterization

- [ ] Yes
- [x] No

Current-voltage characteristics were recorded in accordance with the procedure reported in the manuscript and in the Methods. No specific protocol for preconditioning was applied.

No preconditioning is required before characterization.

Stability of the J-V characteristic
Verified with time evolution of the maximum power point or with the photocurrent at maximum power point; see ref. 7 for details.

- [x] Yes
- [ ] No

Maximum power point tracking was conducted, as reported in the manuscript (Fig. S21b).

Not applicable since the highlight of this work are new concept of perovskite inks formulation.

3. Hysteresis or any other unusual behaviour

Description of the unusual behaviour observed during the characterization

- [x] Yes
- [ ] No

Very low hysteresis was observed upon altering the scan direction is reported in the manuscript (Fig. 2, 5).

Not applicable since the highlight of this work are new concept of perovskite inks formulation.

Related experimental data

- [x] Yes
- [ ] No

The manuscript provides current density - voltage (JV) plots in both forward and backward direction (Fig. 2f, Fig. 5a,b, Fig. S21).

Not applicable since the highlight of this work are new concept of perovskite inks formulation.

4. Efficiency

External quantum efficiency (EQE) or incident photons to current efficiency (IPCE)

- [x] Yes
- [ ] No

IPCE spectra were recorded, as reported in Fig. 2, Fig. S15, S17.

Explain why this information is not reported/not relevant.

A comparison between the integrated response under the standard reference spectrum and the response measure under the simulator

- [x] Yes
- [ ] No

The integrated Jsc from IPCE spectra is consistent with the Jsc from JV measurements, as detailed in the manuscript (Fig. 2e, Fig. S15, S17).

Explain why this information is not reported/not relevant.

For tandem solar cells, the bias illumination and bias voltage used for each subcell

- [ ] Yes
- [x] No

State where this information can be found in the text.

Not applicable as no tandem solar cells are reported in this work.

5. Calibration

Light source and reference cell or sensor used for the characterization

- [x] Yes
- [ ] No

The light source was a 300-W Xenon lamp (Oriel) equipped with a SchottK113 Tempax sunlight filter (Praezisions Glas & OptikGmbH) to match the emission spectrum of the lamp to the AM1.5G standard. Before each measurement, the exact light intensity was determined using a calibrated Si reference diode equipped with an infrared cut-off filter (KG-3, Schott), as detailed in the Materials and Methods (Photovoltaic performance measurements).

Explain why this information is not reported/not relevant.

Confirmation that the reference cell was calibrated and certified

- [x] Yes
- [ ] No

The Si reference cell was calibrated and certified by Newport Corporation PV Lab, Bozeman, MT, USA, as detailed in the Methods.

Explain why this information is not reported/not relevant.

Calculation of spectral mismatch between the reference cell and the devices under test

- [x] Yes
- [ ] No

The spectral mismatch between our simulator and the AM 1.5 solar source was insignificant as the integrated current densities estimated from the IPCE spectra were in good agreement with the values obtained from the current density - voltage (J-V) curves as detailed in the manuscript. Spectra mismatch factor of 1 was used.

Explain why this information is not reported/not relevant.

6. Mask/aperture

Size of the mask/aperture used during testing

- [x] Yes
- [ ] No

All measurements were conducted using a non-reflective metal mask with an aperture area of 0.16 cm2 for small cells and 53 cm2 for modules to cover part of the active area of the device and avoid stray light capturing by our device, as detailed in the Materials and Methods (Photovoltaic performance measurements).

Explain why this information is not reported/not relevant.

Variation of the measured short-circuit current density with the mask/aperture area

- [ ] Yes
- [x] No

State where this information can be found in the text.

We haven't measured the cells with apertures of different sizes.

7. Performance certification

Identity of the independent certification laboratory that confirmed the photovoltaic performance

- [ ] Yes
- [x] No

State where this information can be found in the text.

No certification are conducted in this work since the no record efficiency is reported. And the highlight of this work are new concept of perovskite inks formulation.

A copy of any certificate(s)
Provide in Supplementary Information

- [ ] Yes
- [x] No

State where this information can be found in the text.

Not applicable as we have not yet certified our devices by an independent accredited laboratory.

8. Statistics

Number of solar cells tested

- [x] Yes
- [ ] No

At least 10 devices for each condition were tested, as reported (Table 1, Fig. S7, S16, S18, and S21).

Explain why this information is not reported/not relevant.

Statistical analysis of the device performance

- [x] Yes
- [ ] No

Histograms of efficiency for the devices are reported (Table 1, Fig. S7, S16, S18, and S21).

Explain why this information is not reported/not relevant.

9. Long-term stability analysis

Type of analysis, bias conditions and environmental conditions
For instance: illumination type, temperature, atmosphere humidity, encapsulation method, preconditioning temperature

- [x] Yes
- [ ] No

The long-term stability analysis is detailed in the manuscript with respect to the type of analysis, illumination, bias, and environmental conditions (involving temperature and atmosphere humidity), as well as the exclusion of encapsulation, which is detailed in the manuscript (Fig. S8 and S22)

Explain why this information is not reported/not relevant.
